# The Interaction of RNA Helicase DDX3 with HIV-1 Rev-CRM1-RanGTP Complex during the HIV Replication Cycle

**DOI:** 10.1371/journal.pone.0112969

**Published:** 2015-02-27

**Authors:** Seyed Hanif Mahboobi, Alex A. Javanpour, Mohammad R. K. Mofrad

**Affiliations:** 1 Molecular Cell Biomechanics Laboratory, Departments of Bioengineering and Mechanical Engineering, University of California, Berkeley, California, United States of America; 2 Physical Biosciences Division, Lawrence Berkeley National Lab, Berkeley, California, United States of America; International Centre for Genetic Engineering and Biotechnology, ITALY

## Abstract

Molecular traffic between the nucleus and the cytoplasm is regulated by the nuclear pore complex (NPC), which acts as a highly selective channel perforating the nuclear envelope in eukaryotic cells. The human immunodeficiency virus (HIV) exploits the nucleocytoplasmic pathway to export its RNA transcripts across the NPC to the cytoplasm. Despite extensive study on the HIV life cycle and the many drugs developed to target this cycle, no current drugs have been successful in targeting the critical process of viral nuclear export, even though HIV’s reliance on a single host protein, CRM1, to export its unspliced and partially spliced RNA transcripts makes it a tempting target. Due to recent findings implicating a DEAD-box helicase, DDX3, in HIV replication and a member of the export complex, it has become an appealing target for anti-HIV drug inhibition. In the present research, we have applied a hybrid computational protocol to analyze protein-protein interactions in the HIV mRNA export cycle. This method is based on molecular docking followed by molecular dynamics simulation and accompanied by approximate free energy calculation (MM/GBSA), computational alanine scanning, clustering, and evolutionary analysis. We highlight here some of the most likely binding modes and interfacial residues between DDX3 and CRM1 both in the absence and presence of RanGTP. This work shows that although DDX3 can bind to free CRM1, addition of RanGTP leads to more concentrated distribution of binding modes and stronger binding between CRM1 and RanGTP.

## Introduction

**Fig 1:**
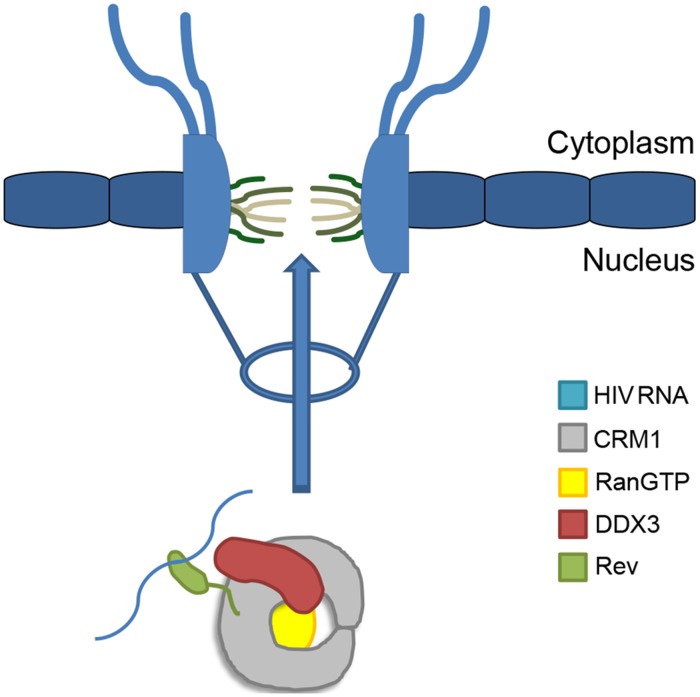
Cartoon schematic of HIV-1 mRNA export complex and nuclear pore complex.

The human immunodeficiency virus (HIV) is a well-known pandemic lentivirus responsible for millions of deaths annually worldwide, particularly in developing and third-world countries [1]. Drugs exist to target nearly every aspect of the viral replication cycle, but treatment aggressiveness is limited by the very potent and potentially dangerous side effects of many of the drugs used. Despite extensive study on the HIV-1 life cycle and the many drugs developed to target this cycle, no current drugs have successfully targeted the critical process of viral nuclear export. HIV’s reliance on a key host protein, CRM1 (also known as XPO1 or Exportin-1), to export its unspliced and partially spliced RNA transcripts makes it a tempting target. HIV Regulator of Virion (Rev) escorts HIV-1 transcripts by recruiting CRM1 (see Fig. 1) and binding to a highly structured region present in all unspliced and partially spliced HIV transcripts, the Rev response element (RRE). Multiple Rev molecules oligomerize cooperatively onto the RRE through several contacts onto the RRE from each Rev to generate a Rev-RRE ribonucleoprotein [2,3]. Targeting of the RRE has been attempted but proven unsuccessful [4,5,6,7]. Targeting the binding of CRM1 to Rev also has been unsuccessful due to lack of detailed structural information [8,9]. Therefore, developing a method that interferes with viral replication at this step would be extremely valuable; however, before this can be considered, the binding interactions between members of the HIV-1 Rev export complex must be elucidated.

The CRM1 export pathway is a delicate target, as it exports numerous cellular proteins and RNA subtypes [10], thereby affecting many cellular pathways. Notably, Leptomycin B, an antifungal shown to permanently modify and disable CRM1 [11], has been demonstrated *in vitro* to inhibit HIV-1 replication in human monocytes [12]. However, because it negatively affects all CRM1-mediated cargo export, it is highly toxic to human cells and not therapeutically applicable. Clearly, while inhibition of CRM1-mediated viral genome export has potentials for combating HIV, complete suppression of the CRM1-export pathway is not an option, and less straightforward approaches must be sought.

Multiple proteins have been implicated in facilitating CRM1 export of HIV-1 Rev cargo through the nuclear pore complex (NPC) as export cofactors. Yedvalli et al. demonstrated that DDX3, a DEAD-box RNA helicase, can bind to CRM1 [13]. Critically, knockdown of DDX3 was shown to strongly inhibit HIV-1 replication [13] without inducing apoptosis [14]. While DDX3 may play a role in CRM1-dependent export of HIV-1 RNA, it does not appear to be necessary for CRM1-dependent export of other cargo, such as IκBα [13]. Yedvalli and colleagues also indicated that DDX3 is specifically active for Rev and RRE-containing mRNAs but not for non-RRE-containing mRNAs. While they argue that DDX3 is a cofactor in nuclear export, this role remains uncertain [15], with critiques suggesting that DDX3 may associate with the HIV-1 Rev-CRM1 export complex at a later step of transport.

These recent findings implicating DDX3 in HIV-1 replication have made it an appealing target for anti-HIV drug inhibition. Indeed, some groups have already begun developing compounds to inhibit various functions of DDX3, such as its RNA helicase [16,17] or ATPase activities [17,18]. These studies have successfully demonstrated as a proof-of-concept that certain classes of compounds targeting DDX3, some of which have been recently patented [19], can inhibit HIV-1 viral replication. Yet, some of the drugs still exhibit significant toxicity, possibly due to off-target effects, necessitating further refinement. Thus, it is crucial to gain deeper insight into the structural interactions between DDX3 and CRM1 in order to design stronger drug compound candidates. Computational docking analysis has the potential to offer such insight about protein-protein interactions. A recent work has provided some insight into the binding between CRM1 and DDX3 using evolutionary analysis and docking [20]; however, this was performed in the absence of Rev and RanGTP. Additionally, their proposed DDX3-binding region on CRM1 overlaps with the location of Rev and CRM1 interaction [9]. In order to gain deeper insight into the binding regions, docking of DDX3 to CRM1 bound to Rev and RanGTP can be performed. Using more rigorous computational approaches with higher resolutions, a deeper understanding of the detailed dynamics of this interaction can be gained.

An important issue regarding binding of DDX3 to CRM1 is the uncertainty in the sequence of events occurring throughout the formation and disassembly of HIV-1 mRNA export complex. One recently proposed structural model suggests a cooperative CRM1-export complex assembly process in which binding of RanGTP invokes large conformational changes in CRM1 leading to a more exposed nuclear export signal (NES) binding cleft and much stronger affinity with NES cargo [21]. This problem is further complicated by DDX3’s RanGTP-independent binding to CRM1. Thus it is unclear at what stage DDX3 comes into play in the formation of the HIV-1 mRNA-Rev-CRM1 export complex.

A complete understanding of the Rev-CRM1-RanGTP-DDX3 export complex has proven to be difficult to obtain experimentally. The creation of such a complete structure, coupled with a clearer understanding of the binding mechanisms, could lead to a significant advancement in the understanding of the HIV-1 RNA-Rev export pathway, and would open the door to future studies. In particular, a full comprehension of the binding dynamics between Rev, CRM1, and DDX3, can aid in future drug development and enable researchers to finally tackle the onerous problem of attacking the viral pathway itself. Construction of a viral export complex consisting of HIV-1 Rev, CRM1-RanGTP, and DDX3 using a computational approach will elucidate the key binding site locations between DDX3 and CRM1 and whether there is cooperativity in the binding of these components in the formation of the export complex.

In the present research, the initial candidates for CRM1-DDX3 binding were determined by protein-protein docking. According to the CAPRI structure prediction contest, this method has experienced considerable progress in the past decade [22,23]. Yet, none of the automated docking servers is able to claim its best solution as the native complex in all test cases [24]. In order to overcome this issue, previous studies suggested using a combination of different docking solutions by consensus scoring [25,26] or post-processing using molecular dynamics (MD) simulation [27]. In this research, we use a combination of docking results obtained from three of the most successful docking servers followed by molecular dynamics equilibration. MD simulation results describing the stability and strength of binding replace the original docking scores for assessment of complex candidates. Computational alanine scanning [28] is used for analysis of key interfacial residues. Some other bioinformatics approaches including hot spot prediction and conservation analysis are also performed to examine their correlation with the simulation results.

## Methods

**Fig 2:**
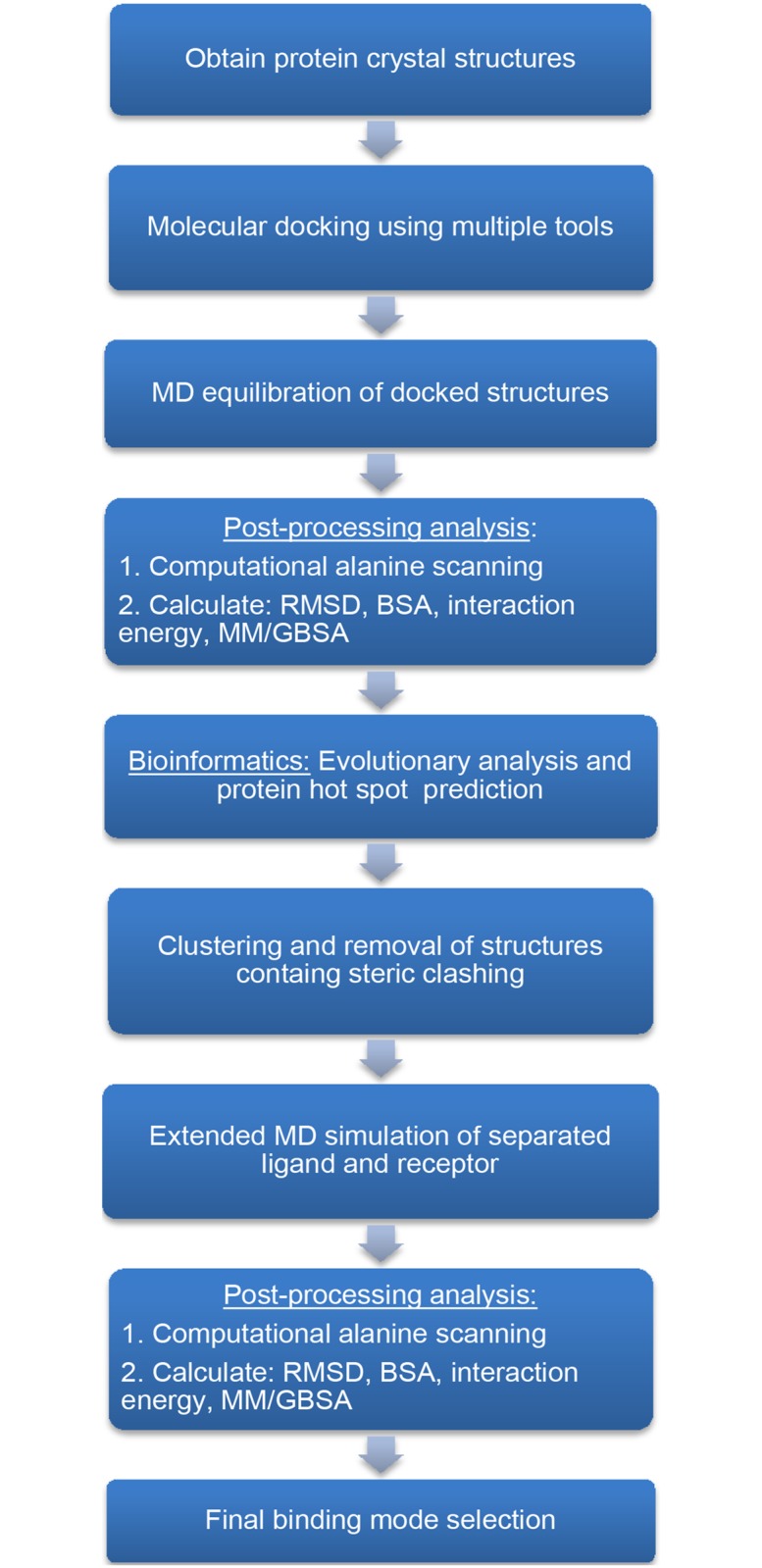
Flowchart of the hybrid computational protocol used for protein-protein binding mode prediction.

Although docking approaches have been traditionally developed for identifying the binding of small molecules (mostly drugs) to large proteins, some recent docking approaches have proven their ability in predicting the binding between proteins. However, due to limitations in protein-protein docking algorithms, one cannot rely solely on binding modes resulting from docking. On the other hand, *de novo* reconstruction of large protein complexes based on pure MD simulation is beyond current computational resources. This calls for a hybrid approach that can leverage the capabilities of multiple methods and tools. A similar protocol, binding estimation after refinement (BEAR), was developed for rapid virtual screening of small ligands using docking, short MD, and scoring calculation using MM/PBSA and MM/GBSA [29]. This work will mainly involve a combination of bioinformatics, docking and MD. Also, free energy analysis will be applied for evaluation of binding strength. Fig. 2 outlines an overview of the protocol.

### Protein Crystal Structures

Crystal structures of DDX3X (PDB ID: 2I4I), herein referred to as DDX3, and CRM1 bound to Snurportin-1 and RanGTP (PDB ID: 3NBZ) or bound to just Snurportin-1 (PDB ID: 3GB8) were obtained from the Protein Databank. All of Snuportin-1 was removed except the NES region from both CRM1 crystal structures. Note that 3NBZ contains a Snuportin-1 molecule with the NES region from HIV-1 Rev. The missing H atoms were added to the structures and they were minimized before docking.

### Molecular Docking

To obtain a list of potential CRM1-DDX3 binding modes, a series of docking simulations were carried out by binding DDX3 with the two CRM1 complexes. Docking was carried out using three webserver tools: ClusPro2.0 [30], GRAMM-X [31], and FireDock [32]. These servers were selected as they were some of the most successful based on the CAPRI benchmarks [23]. Broadly, these docking tools use an algorithmic approach to explore all potential geometries of binding while treating the interacting proteins as rigid bodies. FireDock performs an additional refinement procedure by introducing side-chain flexibility to the rigid-body docked structures and performing a side-chain optimization. Subsequently, Monte Carlo energy minimization is performed and a final ranking is obtained based on a binding score. No interface constraints were used with GRAMM-X. Also, PatchDock [33] was used first to generate a primary list of docked structures, and then these structures were passed to FireDock for further refinement. The top 10 docked structures from each of these tools (30 bound structures for each CRM1 complex; 60 total structures) were gathered for further analysis.

### Molecular Dynamics Simulation

Molecular dynamics (MD) simulation was performed using each docked structure to further refine the binding mode. MD models were built using NAMD 2.9 [34] and the CHARMM27 force field [35,36]. Protein manipulations, measurements, and water box addition were done with VMD1.9.1 and the included plugins [37].

This system was placed in a water box with the TIP3P water molecule, a periodic simulation cell with a 10 Å margin and Na^+^ and Cl^-^ counter-ions at the concentration of 150mM. In all simulations, Particle Mesh Ewald [38] was used for electrostatic energy calculation. Total atom numbers varied from 200,000 to 230,000 for different cases.

In order to conserve computation resources the bonds between hydrogen and larger atoms were held at fixed length, and thus, a timestep of 2 fs was used. The default multiple timestepping method of NAMD was used [39], with 2 fs step for bonded force evaluation, 2 fs for nonbonded forces, and 4 fs for long-range electrostatics. Pressure was regulated at 1 atm using Langevin piston [40] with a period of 100 fs and damping timescale of 50 fs, and Langevin damping factor of 1 fs^-1^. Three independent simulations, each 10 ns long at 310 K, as well as preliminary minimization were performed for each docked structure. The second half (5 ns) of the simulation time is considered as the production part.

### Binding Analysis and Free Energy Calculation

Energetic and structural analysis was performed subsequent to the simulations. Specifically, root mean square deviation (RMSD), buried surface area (BSA), interaction energy between CRM1 and DDX3, and MM/GBSA were calculated.

Molecular Mechanics/Generalized Born Surface Area (MM/GBSA) is an approximate free energy calculation method that uses a combination of molecular mechanics energy and implicit solvation models. It is much more accurate than conventional docking scores yet more computationally expensive. However, it is faster and more convenient than rigorous free energy calculation methods such as umbrella sampling or alchemical approaches [41,42,43]. This method and its counterpart based on Poisson-Boltzmann approximation (MM/PBSA) have been successfully utilized to compare conformational stabilities and binding free energies in a variety of cases including nucleic acids structures [41], protein folding [44], protein-ligand complexes [45] and computational mutagenesis [46]. MM/GBSA is categorized as an end-point method for free energy calculation and is derived from direct calculation of various components of free energy including bonded, electrostatic and vdW energies, polar and non-polar desolvation free energies with addition of conformational entropy. Because we have similar ligands in different MM/GBSA calculations, dropping the entropic term can still give us reasonable results if we just focus on the relative free energies. Similarly, the BEAR protocol also excludes the entropic term from this calculation. This way we can also save a lot of computational resources. It must be noted that even inclusion of entropy portion for protein-protein complexes will not give reliable absolute free energy values [47,48]. Parameter set GB^OBC^II was used for calculation of GB term [49]. Surface tension coefficient was set at 0.005 in nonpolar solvation energy term. We chose the last 5ns from each simulation and uniformly picked 100 sampling points for the calculation of different MM/GBSA terms.

Computational alanine scanning (CAS) [50] was performed using the webserver Robetta [28] in order to ascertain interfacial residues critical in maintaining stability between CRM1 and DDX3. Like traditional experimental alanine scanning, Robetta CAS server mutates interfacial residues to alanine and the change in free energy of binding is calculated using a linear free energy function. Robetta reports a list of “hot spots” consisting of residues that would significantly destabilize the interface of two bound proteins if mutated to alanine with the associated change in free energy. CAS was performed for all 60 docked structures. Using CAS, a list of interfacial residues is compiled and is the basis of the predicted binding mode for CRM1 and DDX3.

Two additional bioinformatics tools were used for further validation, SPPIDER [51] and ConSurf [52]. SPPIDER is a webserver that can predict functional residues at protein-protein interaction sites based on a consensus-classifier. The two different crystal structures of CRM1 and DDX3 were submitted to the SPPIDER webserver and the list of putative interface residues was complied. The settings ‘SPPIDER I’ and a tradeoff of 0.3 were chosen. Another tool, ConSurf, gives a measure of how evolutionarily conserved the positions of amino acids in a given protein are based on phylogenetic relations of homologous protein sequences. Again, the same crystal structures of CRM1 and DDX3 were submitted individually to the ConSurf webserver. The multiple sequence alignment was first generated from ConSurf using the UNIREF-90 protein database [53] and CSI-BLAST homolog search algorithm with 3 iterations and an E-value cutoff of 0.0001. A Bayesian calculating method with a Jones-Taylor-Thornton [54] evolutionary substitution model was used to calculate the conservation scores.

### Clustering and Extended MD

Following MD simulation, it was noticed that there was redundancy within the 60 docked structures as a result of using multiple docking tools. Thus, clustering was performed to collapse the 60 docking structures to a set of non-redundant binding modes. A hierarchical RMSD-based clustering algorithm was adapted from ClusPro2.0. VMD was first used to perform the necessary RMSD calculations. For two given docked structures, the CRM1 structures were aligned. Then, the RMSD between the two DDX3 molecules was calculated. This procedure was performed pairwise for all 60 structures to generate a 60 x 60 RMSD matrix. Clusters are then generated by selecting all docked structures that are below a cutoff of 30 Å from the reference docked structure, designated the cluster center. Then, the clusters are sorted by the number of members in each cluster. After finding the largest cluster, all of its members will be removed from the list and the next largest cluster will be determined. This procedure is repeated for all subsequent clusters. After clustering, structures that contained a DDX3 bound to a location that sterically prohibited the binding of RanGTP (in the case of 3GB8) or RanBP1 (PDB ID: 4GMX; both 3GB8 and 3NBZ cases) were removed. For each cluster with more than 4 members, the most structurally and energetically stable docked complex was selected for extended MD simulation. In addition to these docked complexes, the most energetically and structurally stable complexes not found within the top clusters were also selected for extended simulation. This is so that any structurally stable complex not clustered well would still be included.

After selecting these top docked structures, a new set of 50 ns extended MD simulations was performed following 10000-step minimization. For each of these structures, DDX3 was moved 10 Å away from CRM1 surface. All simulation conditions are the same as the previous 10 ns simulations. Energetic and structural analysis was performed once again after these extended simulations consisting of calculating RMSD, interaction energy, MM/GBSA, and BSA. Additionally, CAS was performed again using Robetta.

### Flexible Multi-Domain Docking

Among the methods used for protein-protein docking, few can handle more than two interacting molecules at a time. Moreover, most of these methods are restricted to symmetric homomeric complexes. HADDOCK [55] is one of the few tools able to handle simultaneous multi-body docking. Moreover, in some circumstances one of the interacting proteins is composed of two separate domains connected by a highly flexible linker. In such cases we may observe global changes including large-scale domain motions like hinge and shear. The flexible multi-domain docking (FMD) feature of HADDOCK multi-body docking enables examining the highly flexible linker between domains while allowing them to independently dock on the other agent [56]. Our particular system can benefit from this utility since the two lobes of DDX3 are free to explore different conformations especially when DDX3 binds to RNA. FMD complemented our main protocol to ensure covering other possible DDX3 conformations and to let it open or close freely upon encountering the host (CRM1). WeNMR was used as the computational resource for running the HADDOCK jobs [57].

In the flexible multi-domain docking (FMD) method, the protein structure is divided into domains separated by flexible linkers between each pair. The location of the flexible hinge region can be determined using an elastic network model. Hingeprot was used to identify the separation point between the domains of the flexible structure [58]. HADDOCK requires a list of interaction restraints to start with. The knowledge about interactions should ideally come from unambiguous experimental studies. Presently, however, such experimental data are scarce for many biological complexes including the one we deal with. Hence, we must identify the potential interacting residues with the help of predictor servers. CPORT was used here to identify the potential interacting residues [59]. CPORT gathers the outcomes from other prediction servers and makes a collective list of the predicted interfacial residues outcomes. It must be taken into account that the list of residues for each protein is independent of other interacting proteins and is derived solely based on each protein structure. The list of suggested interfacial residues was then used to prepare the ambiguous interaction restraints as input to the HADDOCK server.

The flexible molecule, DDX3X in the present work, is divided into two separate pieces. Hence, the connectivity between the separated domains must be defined and maintained throughout the docking procedure. This can be implemented as unambiguous distance restrains between the C- and N-termini. The server returns the list of solutions sorted based on the highest scores, where a score is defined as the interaction energy penalized by violation of distance constraints. The candidates are also clustered according to their proximity to each other. From this list, only solutions that satisfy the distance constraint between the two domains will be considered feasible candidates. Also, possible overlap with other interacting molecules with the receptor must be considered to filter out undesirable candidates.

## Results

### Molecular Docking of DDX3 to CRM1

The ultimate objective is to predict the binding mode of the CRM1-NES-RanGTP-DDX3 protein complex and elucidate residues strongly implicated in binding between DDX3 and the CRM1 export complex. Due to lack of detailed, experimental information regarding the binding mode of DDX3 to CRM1 export complex, molecular docking was used in order to obtain a sample of possible binding modes. With this information, it will then be possible to perform targeted, systematic MD simulations with DDX3 and the CRM1 export complex.

**Fig 3:**
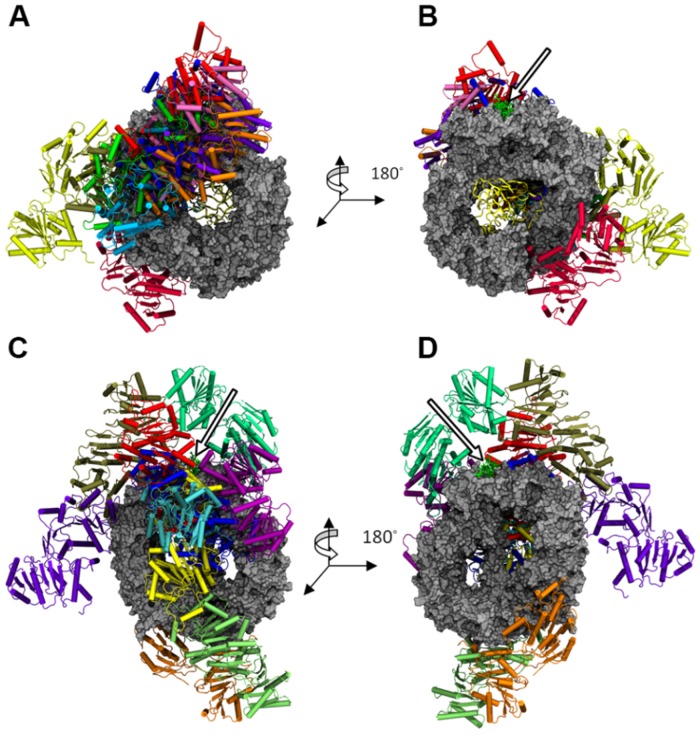
Superposition of the top 10 (for each host structure) ClusPro2.0 docked structures. The structures are shown for (A) 3NBZ (w/RanGTP) back side (B) 3NBZ front side, and (C) 3GB8 (w/o RanGTP) back side, and (D) 3GB8 front side. CRM1 is shown in silver and RanGTP is the yellow ribbon structure. The arrow points to the Rev-NES peptide.

To assess whether DDX3 has a different binding mode with CRM1 as a function of RanGTP, we first docked DDX3 to two different forms of the CRM1 export complex. The first form contains CRM1 bound to an NES peptide as well as RanGTP while the second form is only bound to NES. Three separate servers (ClusPro2.0, GRAMM-X, FireDock) were used in order to avoid any biases in a given docking algorithm and therefore obtain a varied set of sample binding modes. The top 10 ranked docking structures were selected from each server. The key binding locations for DDX3 on CRM1 were, first, near the N-C terminal junction, second, near the NES peptide, third, distributed somewhere along the rim of CRM1, and fourth, on the back of CRM1 (i.e., the side opposite where Ran binds). Examining the top 10 results from ClusPro, DDX3 was docked 8 times in some position radiating from the center of the back of CRM1 and twice along the bottom rim of CRM1 from 3NBZ (Fig. 3). Five of the docked DDX3 molecules on the back of CRM1 are within close proximity to the NES peptide. Conversely, a different distribution of binding locations for DDX3 was observed when it was docked to CRM1 from 3GB8, which lacks the presence of RanGTP. Namely, there was an even distribution of DDX3 molecules docked all along the rim of CRM1. Interestingly, the top 10 GRAMM-X results using CRM1 from 3NBZ mimicked the same general DDX3 binding distributions as seen with ClusPro2.0, with only one DDX3 molecule far from the NES (S1 Fig.). Similarly, there was no discernible trend with the DDX3 binding distribution using CRM1 from 3GB8, as seen with ClusPro2.0. On the other hand, this behavior was not noticed with FireDock (S2 Fig.). In the 3NBZ case, there was an arbitrary distribution of DDX3 around CRM1, while the 3GB8 case had half of the DDX3 molecules bound close to the NES or on the opposite side of CRM1, at the bottom. With this, we have obtained a set of 30 protein complexes containing CRM1-NES-RanGTP-DDX3 and 30 protein complexes containing CRM1-NES-DDX3.

### Molecular Dynamics Simulation of Bound CRM1-DDX3 complexes

Docking provides a distribution of potential binding modes but lacks information regarding the stability of the binding modes. Additionally, most docking tools do not allow of large conformational changes commonly found in protein-protein binding. To determine the specific binding residues accurately, further refinement was necessary to each of the docked protein complexes. Thus, all-atom molecular dynamics (MD) simulation was performed to minimize and equilibrate the protein complexes.

After simulation, several system attributes/quantities were calculated for each of the 60 complexes (S3 Fig.). First, the root-mean-square deviation (RMSD) of the protein complex was monitored to see if the system reached a steady value, indicating an equilibrium was reached (see S4 Fig. for some sample cases), and the RMSD was computed for the last 5 ns (i.e., the production part) of the simulation. Second, the interaction energy between DDX3 and CRM1 was determined to gauge the strength of binding. Next, the change in buried surface area (BSA) was measured to help define a potential binding event. Finally, the molecular mechanics/generalized-Born implicit solvent (MM/GBSA) was calculated for an estimation of the binding free energy. Recall that the entropic term was not included in our calculations and thus can only be used to compare free energies amongst the 60 docked structures.

The values for each of these four quantities are listed and ranked in Table 1 for all 60 docked complexes. Broadly, the docked complexes from ClusPro2.0 using 3NBZ had the lowest RMSD, strongest interaction energy, largest MM/GBSA and greatest BSA. This is the same for the 3GB8 case except GRAMM-X generally had lower RMSD and the highest BSA. The results from ClusPro2.0 indicated its docked structures were more stable with stronger binding than other servers may be due in part because of the large number of solutions involved in the clustering process. In other words, the outcome is the representative of large number of strong complexes. The average results from the servers comparing 3NBZ and 3GB8 are similar (S1 Table). However, ClusPro2.0 results could possibly be weighted more due to its clustering process. Moreover, ClusPro2.0 achieved top results from CAPRI. Considering this, when examining the 3NBZ and 3GB8 ClusPro2.0 results, the 3NBZ docked structures achieved much stronger binding. There did not seem to be any discernible correlation between the docking tool rankings and the rankings of the calculated quantities in Table 1. This can be expected because each server has their own ranking criteria that may not include these four calculated quantities seen in Table 1. Among the four quantities, there seemed to be some observable correspondence among the rankings of a given docked complex. Specifically, the rankings for interaction energy, MM/GBSA, and BSA seemed to trend with one another. For example, a docked structure that had a high rank for interaction energy, such as (3NBZ) ClusPro #6, tended to also have a high rank for MM/GBSA and BSA. Conversely, a low rank for interaction energy correlated with a low rank for MM/GBSA and BSA.

**Table 1:** Calculated structural and energetic quantities for the 60 docked structures with rankings.

Server	Server Rank	RMSD (Å)	CRM1-DDX3 Int. Energy (kcal/mol)	(CRM1+RanGTP)-DDX3 Int. Energy (kcal/mol)	MM/GBSA (kcal/mol)	BSA (Å^2^)
3NBZ						
**ClusPro**	1	3.37 ± 0.23 (8)	-848.8 ± 29.0 (11)	-897.7 ± 46.5 (13)	-788.3 ± 14.3 (38)	2917.2 ± 222.8 (22)
	2	4.70 ± 0.49 (43)	-564.8 ± 27.9 (25)	-564.8 ± 27.9 (30)	-838.9 ± 4.1 (8)	2724.6 ± 39.7 (26)
	3	3.33 ± 0.25 (7)	-706.4 ± 95.7 (16)	-926.5 ± 98.5 (9)	-809.3 ± 18.6 (22)	3683.5 ± 509.9 (7)
	4	4.00 ± 0.08 (28)	-894.6 ± 12.6 (8)	-1229.8 ± 2.8 (2)	-821.3 ± 8.1 (15)	3480.2 ± 286.1 (11)
	5	3.28 ± 0.21 (6)	-1040.0 ± 57.0 (4)	-1041.0 ± 48.4 (8)	-850.7 ± 7.6 (5)	4297.4 ± 80.1 (4)
	6	3.10 ± 0.09 (3)	-917.0 ± 95.7 (6)	-916.3 ± 98.4 (10)	-851.2 ± 29.1 (4)	4368.9 ± 453.0 (3)
	7	2.84 ± 0.12 (1)	-1074.1 ± 51.8 (3)	-1371.7 ± 22.1 (1)	-869.7 ± 6.4 (1)	5162.0 ± 220.2 (1)
	8	4.26 ± 0.72 (34)	-449.4 ± 40.9 (36)	-622.7 ± 35.1 (24)	-818.3 ± 9.0 (16)	3020.5 ± 308.5 (18)
	9	3.64 ± 0.09 (17)	-645.1 ± 113.5 (18)	-649.1 ± 110.0 (21)	-808.8 ± 13.3 (23)	2656.7 ± 364.3 (29)
	10	3.18 ± 0.24 (4)	-1006.7 ± 78.8 (5)	-1221.6 ± 68.7 (3)	-840.6 ± 16.1 (7)	4766.5 ± 65.6 (2)
**FireDock**	1	3.65 ± 0.24 (18)	-186.4 ± 44.0 (57)	-337.1 ± 60.0 (48)	-751.2 ± 3.7 (60)	1655.2 ± 95.0 (51)
	2	3.25 ± 0.20 (5)	-734.3 ± 54.5 (15)	-734.3 ± 54.5 (18)	-836.1 ± 8.9 (9)	3310.6 ± 76.2 (14)
	3	4.27 ± 0.30 (35)	-610.2 ± 63.8 (20)	-1086.0 ± 76.5 (7)	-804.9 ± 15.5 (25)	3038.9 ± 327.1 (17)
	4	4.72 ± 0.34 (45)	-159.4 ± 13.0 (59)	-159.4 ± 13.0 (60)	-769.5 ± 4.1 (55)	1468.6 ± 91.9 (56)
	5	3.92 ± 0.20 (23)	-339.6 ± 38.6 (43)	-389.8 ± 38.0 (42)	-777.9 ± 8.0 (48)	1598.1 ± 138.3 (53)
	6	3.98 ± 0.57 (27)	-396.3 ± 45.0 (38)	-404.5 ± 40.0 (41)	-783.2 ± 3.5 (44)	2383.2 ± 287.4 (36)
	7	3.51 ± 0.24 (12)	-267.0 ± 22.0 (52)	-267.0 ± 22.0 (56)	-765.0 ± 5.0 (58)	1616.4 ± 148.8 (52)
	8	3.93 ± 0.37 (25)	-170.9 ± 45.0 (58)	-387.5 ± 44.5 (43)	-778.8 ± 5.2 (46)	1214.9 ± 80.5 (60)
	9	5.56 ± 0.79 (57)	-17.9 ± 6.8 (60)	-464.4 ± 35.2 (37)	-798.8 ± 7.6 (30)	1341.7 ± 86.3 (58)
	10	5.06 ± 0.23 (53)	-562.4 ± 22.0 (26)	-650.4 ± 15.8 (20)	-803.0 ± 3.8 (27)	2411.9 ± 362.9 (34)
**GRAMM-X**	1	3.53 ± 0.41 (13)	-466.1 ± 15.2 (33)	-907.7 ± 43.2 (12)	-791.2 ± 8.2 (36)	3511.3 ± 326.2 (10)
	2	3.94 ± 0.54 (26)	-302.7 ± 24.1 (49)	-308.1 ± 25.6 (53)	-794.1 ± 10.0 (33)	1823.2 ± 308.3 (47)
	3	4.43 ± 0.38 (39)	-457.0 ± 107.8 (34)	-453.8 ± 104.9 (38)	-788.9 ± 3.0 (37)	2193.1 ± 194.8 (40)
	4	4.44 ± 0.16 (40)	-237.5 ± 53.6 (54)	-237.9 ± 54.0 (57)	-787.1 ± 18.8 (40)	1685.2 ± 230.5 (49)
	5	3.02 ± 0.24 (2)	-359.7 ± 74.4 (40)	-512.7 ± 75.5 (36)	-784.2 ± 3.3 (43)	2966.1 ± 104.6 (21)
	6	4.22 ± 0.63 (33)	-260.2 ± 17.4 (53)	-332.7 ± 13.8 (50)	-768.9 ± 1.6 (56)	2127.4 ± 57.3 (41)
	7	3.74 ± 0.17 (21)	-309.3 ± 33.5 (47)	-310.6 ± 32.6 (52)	-760.6 ± 11.8 (59)	2020.6 ± 213.6 (43)
	8	3.37 ± 0.18 (9)	-736.8 ± 33.0 (14)	-1176.5 ± 28.1 (4)	-796.8 ± 2.0 (32)	3769.3 ± 271.7 (6)
	9	4.71 ± 0.50 (44)	-545.6 ± 41.7 (28)	-596.7 ± 33.7 (26)	-801.3 ± 5.2 (28)	2846.3 ± 171.6 (23)
	10	3.58 ± 0.07 (14)	-293.3 ± 16.2 (50)	-692.1 ± 18.7 (19)	-813.1 ± 0.5 (19)	2766.5 ± 137.1 (25)
3GB8						
**ClusPro**	1	5.06 ± 1.13 (52)	-528.5 ± 41.9 (29)	-528.5 ± 41.9 (33)	-814.0 ± 0.8 (18)	2402.7 ± 48.8 (35)
	2	4.94 ± 0.48 (50)	-527.8 ± 33.0 (30)	-529.2 ± 33.4 (32)	-806.0 ± 8.4 (24)	2113.8 ± 169.4 (42)
	3	3.77 ± 0.47 (22)	-880.5 ± 61.0 (9)	-880.5 ± 61.0 (14)	-818.0 ± 20.3 (17)	3073.5 ± 122.0 (16)
	4	4.67 ± 0.41 (42)	-1164.7 ± 168.0 (1)	-1164.8 ± 168.1 (5)	-835.6 ± 15.1 (10)	2653.7 ± 424.3 (30)
	5	5.59 ± 0.61 (58)	-336.7 ± 64.7 (44)	-336.6 ± 64.8 (49)	-773.0 ± 8.3 (53)	1530.8 ± 118.2 (55)
	6	3.40 ± 0.31 (10)	-585.5 ± 58.3 (22)	-587.5 ± 59.1 (27)	-829.0 ± 3.1 (11)	2678.7 ± 233.4 (28)
	7	3.48 ± 0.40 (11)	-878.1 ± 149.8 (10)	-878.1 ± 149.8 (15)	-825.4 ± 19.6 (13)	3095.4 ± 434.8 (15)
	8	3.74 ± 0.25 (20)	-777.3 ± 11.6 (13)	-777.7 ± 11.8 (17)	-856.7 ± 8.9 (2)	2974.8 ± 167.5 (20)
	9	5.91 ± 0.78 (59)	-324.8 ± 24.9 (45)	-371.3 ± 12.9 (45)	-780.2 ± 2.3 (45)	1942.8 ± 38.0 (45)
	10	4.77 ± 0.22 (47)	-554.8 ± 74.0 (27)	-553.5 ± 75.7 (31)	-809.5 ± 11.8 (21)	2331.1 ± 164.6 (37)
**FireDock**	1	4.06 ± 0.31 (29)	-599.4 ± 45.1 (21)	-599.4 ± 45.1 (25)	-791.6 ± 7.5 (35)	2541.1 ± 135.4 (32)
	2	4.56 ± 0.42 (41)	-622.8 ± 33.5 (19)	-623.2 ± 34.0 (23)	-822.9 ± 7.4 (14)	2295.1 ± 127.7 (38)
	3	4.13 ± 0.19 (31)	-228.4 ± 27.8 (55)	-228.4 ± 27.8 (58)	-785.8 ± 4.3 (41)	1759.9 ± 120.3 (48)
	4	3.70 ± 0.03 (19)	-453.6 ± 49.4 (35)	-453.6 ± 49.4 (39)	-784.5 ± 3.3 (42)	2489.7 ± 186.8 (33)
	5	5.46 ± 0.82 (56)	-419.7 ± 35.2 (37)	-419.7 ± 35.2 (40)	-775.6 ± 7.5 (50)	1543.7 ± 115.2 (54)
	6	5.27 ± 1.12 (54)	-342.7 ± 44.2 (41)	-342.7 ± 44.2 (46)	-767.8 ± 20.4 (57)	1679.4 ± 373.7 (50)
	7	5.03 ± 0.10 (51)	-304.0 ± 77.6 (48)	-304.0 ± 77.6 (54)	-773.2 ± 6.0 (52)	1396.1 ± 266.2 (57)
	8	3.59 ± 0.44 (15)	-579.4 ± 72.3 (24)	-579.4 ± 72.3 (29)	-848.0 ± 16.0 (6)	3362.7 ± 111.4 (13)
	9	5.91 ± 0.46 (60)	-340.3 ± 81.5 (42)	-340.3 ± 81.5 (47)	-810.1 ± 10.6 (20)	1967.2 ± 32.9 (44)
	10	4.30 ± 0.17 (36)	-378.2 ± 74.0 (39)	-378.2 ± 74.0 (44)	-769.8 ± 15.0 (54)	2204.5 ± 350.1 (39)
**GRAMM-X**	1	4.90 ± 0.32 (49)	-318.0 ± 47.4 (46)	-318.0 ± 47.4 (51)	-777.2 ± 9.4 (49)	3019.0 ± 199.7 (19)
	2	4.81 ± 0.43 (48)	-1126.4 ± 105.5 (2)	-1126.4 ± 105.5 (6)	-856.5 ± 6.5 (3)	3873.3 ± 239.9 (5)
	3	4.16 ± 0.52 (32)	-214.6 ± 71.9 (56)	-214.6 ± 71.9 (59)	-773.6 ± 7.1 (51)	1828.7 ± 157.0 (46)
	4	3.92 ± 0.29 (24)	-908.0 ± 60.5 (7)	-907.8 ± 60.5 (11)	-797.7 ± 12.5 (31)	3652.6 ± 200.6 (8)
	5	4.42 ± 0.58 (38)	-798.0 ± 110.7 (12)	-798.0 ± 110.7 (16)	-826.1 ± 10.1 (12)	3511.3 ± 240.9 (9)
	6	3.61 ± 0.88 (16)	-645.3 ± 48.0 (17)	-645.3 ± 48.0 (22)	-787.4 ± 7.3 (39)	3376.8 ± 60.1 (12)
	7	5.30 ± 0.37 (55)	-272.0 ± 43.6 (51)	-272.0 ± 43.6 (55)	-778.5 ± 15.8 (47)	1242.7 ± 200.7 (59)
	8	4.08 ± 0.13 (30)	-522.3 ± 78.5 (32)	-522.3 ± 78.5 (35)	-803.2 ± 21.4 (26)	2818.6 ± 212.3 (24)
	9	4.76 ± 0.50 (46)	-526.6 ± 30.3 (31)	-526.6 ± 30.3 (34)	-799.8 ± 7.1 (29)	2691.1 ± 165.8 (27)
	10	4.35 ± 0.77 (37)	-581.5 ± 55.3 (23)	-581.5 ± 55.3 (28)	-793.2 ± 6.7 (34)	2614.1 ± 146.9 (31)

Numbers in parenthesis indicate the ranking among 60 candidates. Standard errors are derived from the performed triplicate simulations.

Naturally, complexes with lower RMSDs are structurally stable and do not exhibit large conformational changes after binding. The time evolution of RMSD for a complex with low RMSD generally reached a stable value during the equilibration phase of MD simulation (S4 Fig.). Moreover, each of the three simulations exhibited the very similar time evolution over the 10 ns. There were no complexes with proteins that became detached but there was a noticeable spread in RMSD values, allowing us to discern which docked structures were least stable.

Energetic analysis gives another diagnostic with which to ascertain strong and stable binding modes. MM/GBSA gives a measure of binding free energy. As seen in Table 1, there is little spread from the smallest value, 751 kcal/mol, to the largest, 870 kcal/mol. Because the entropic term is neglected due to a lack of computational resources, this term is only used as a relative measure among the 60 docked structures. The interaction energy between CRM1 and DDX3 as well as CRM1+RanGTP and DDX3 are listed separately in Table 1. There is a large range of interaction energy values, from 18 to over 1300 kcal/mol. Notice that there are several cases where the interaction energy is substantially stronger when the interaction between RanGTP are included, such as (3NBZ) FireDock #9 (from-18 to-464 kcal/mol). It is necessary to be cognizant of the fact that only a few residues need be involved in protein-protein binding to achieve a stable interaction. Thus, docked structures with lower interaction energies cannot be ruled out exclusively based on the criteria of interaction energy.

### Evaluating Key Binding Residues with Computational Alanine Scanning, Interfacial Residue Prediction (SPPIDER) and Evolutionary Conservation (ConSurf)

**Fig 4:**
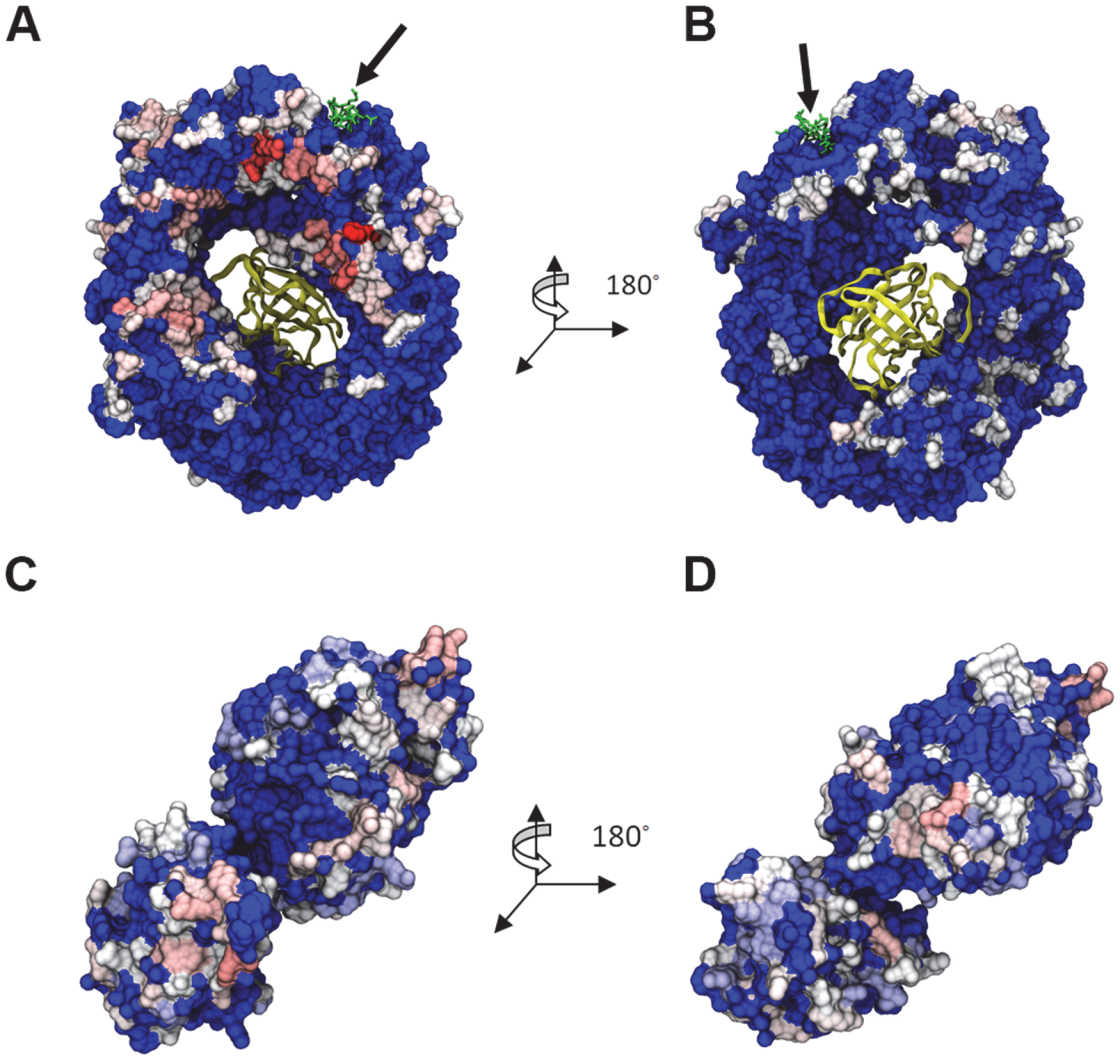
Residues with ΔG_mut→Ala_ >1 kcal/mol highlighted on the molecules from DDX3-CRM1-RanGTP complex. (A) CRM1 back, (B) CRM1 front, (C) and (D) DDX3. Color scale from white (~1 kcal/mol) to red (>2 kcal/mol). Blue indicates 0 kcal/mol. The arrow points to the Rev-NES peptide.

**Fig 5:**
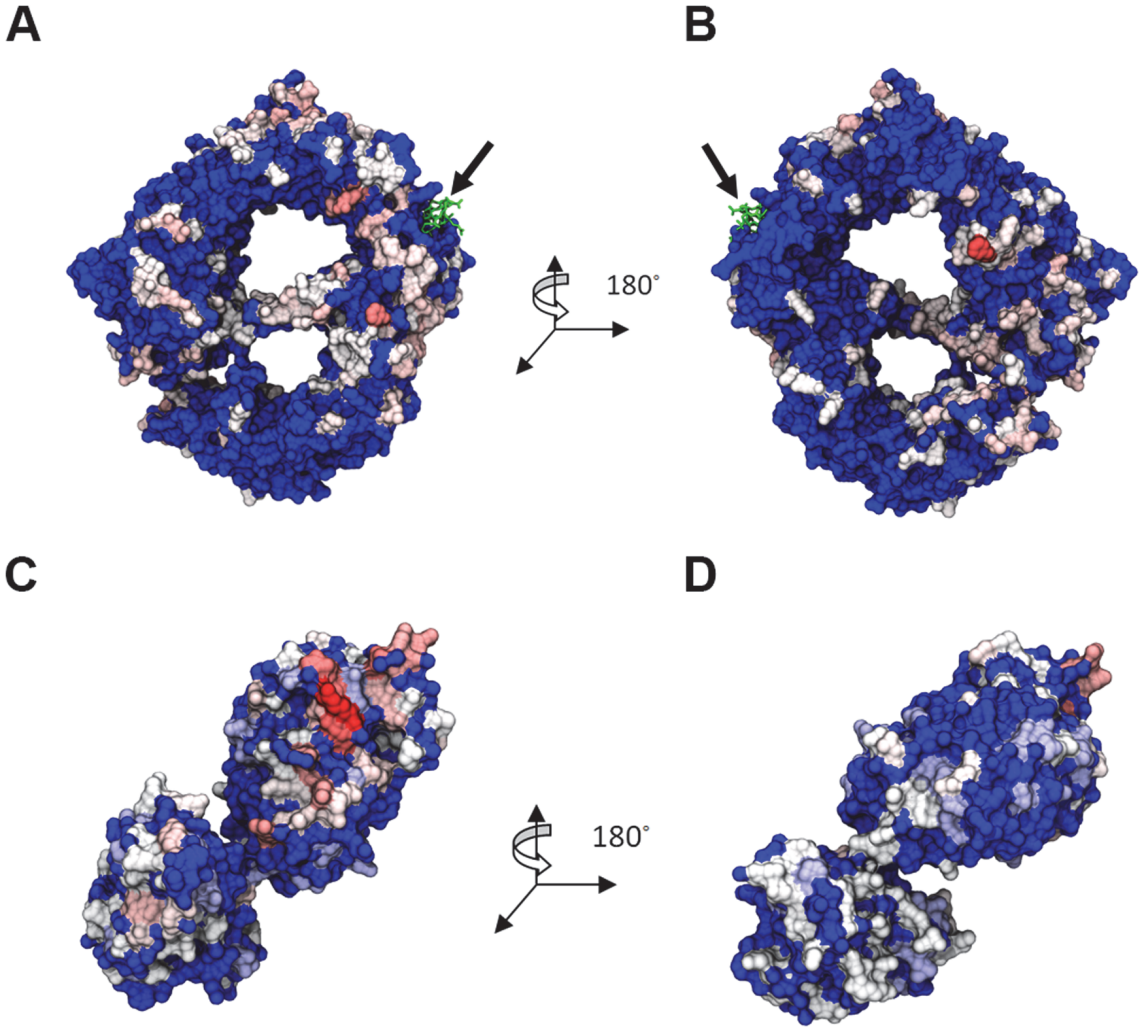
Residues with ΔG_mut→Ala_ >1 kcal/mol highlighted on the molecules from DDX3-CRM1 complex. (A) CRM1 back, (B) CRM1 front, (C) and (D) DDX3. Color scale from white (~1 kcal/mol) to red (>2 kcal/mol). Blue indicates 0 kcal/mol. The arrow points to the Rev-NES peptide.

The interacting residues between CRM1 and DDX3 were then determined using computational alanine scanning. The location of each binding residue for all 30 3NBZ docked structures (Fig. 4) and 30 3GB8 docked structures (Fig. 5) were highlighted by color based on the frequency of appearance. The hottest binding regions for CRM1 from 3NBZ were located on the back of CRM1 and near the NES binding location. CRM1 from 3GB8 instead exhibited binding residues spread all around the structure, front and back, with a hot region below the NES binding location. There was no discernible bias in the binding residues of DDX3 when bound either to CRM1 from 3NBZ or 3GB8.

SPPIDER and ConSurf were then applied to our system. Using SPPIDER, a list of predicted interaction residues was obtained for CRM1 (both from 3NBZ and 3GB8) and DDX3 (S5 Fig.). The predicted interaction sites for CRM1 from 3NBZ are located along the upper rim of CRM1, flanking both sides of the NES binding region, and at the lip protruding on the front face of the bottom of CRM1, underneath Ran (Panels A and B in S5 Fig.). The predicted binding locations on CRM1 from 3GB8 show the same regional localization as with the 3NBZ case but with a lower density of predicted sites (Panels C and D in S5 Fig.). The predicted interface sites on DDX3 occurred around the RNA and ATP binding sites, located in the upper lobule, and on the lower lobule at its interfacial region with the upper lobule (Panels E and F in S5 Fig.).

Evolutionary conservation scores for each residue were mapped onto each amino acid in S6 Fig. The strongest region of conservation on CRM1 from both 3NBZ (Panels A and B in S6 Fig.) and 3GB8 (Panels C and D in S6 Fig.) occurred at the NES binding location and the associated binding location of RanGTP. This is expected as NES containing proteins and Ran are critical binding partners and give functionality to CRM1. Additional strongly conserved binding locations speckle CRM1 in both cases all over the protein. For DDX3, the hottest regions of conservation are the locations of RNA and ATP binding and the regions surrounding these two binding spots (Panels E and F in S6 Fig.).

### Clustering and Extended MD Candidate Selection

To reiterate, the final goal is to predict the most probable CRM1-NES-DDX3 binding mode. At this current stage, we have 60 possible binding modes and it is necessary to systematically reduce the list of docking structures. While the goal of using multiple docking servers is to avoid any bias present in a given docking algorithm, the methods used by each tool is not so distinct such that there will be no overlap in the binding modes among the three servers. Indeed, we observed some redundant binding modes within the set of the docked structures. Thus, structural clustering was necessary to generate a minimal, non-redundant list of docked structures. Briefly, docked structures that had a DDX3 RMSD below a specific cutoff were clustered together. Then, the docked structure that had the best overall RMSD, interaction energy, MM/GBSA, and BSA from each cluster with at least 4 members was selected as representative of the cluster. Prior to this selection, docked structures that had any structural clashing with the binding location of other known CRM1-binding partners, namely RanGTP and RanBP1, were excluded. Recall that one type of the CRM1 binding complexes (3GB8) analyzed does not have RanGTP. As binding of RanGTP is a requirement for nuclear export, DDX3 cannot be docked to this location and any docked structure that has this structural clashing is removed. Additionally, RanBP1 is required for complex disassembly and its binding location on CRM1 must be open. So, any docked structures that have DDX3 positioned in a location that would sterically clash with RanBP1 were also removed from the clustering. After this clustering process, there were four clusters with at least 4 members. The best docked structures in each of these clusters were (3NBZ) GRAMM-X #8, (3NBZ) ClusPro #6, (3GB8) ClusPro #7, (3NBZ) ClusPro #7.

In addition to clustering, the docked structures with the strongest binding based on RMSD, interaction energy, MM/GBSA, and BSA from Table 1 were also selected for further analysis, namely extended MD simulation. These docked structures are (3NBZ) ClusPro #6, (3NBZ) ClusPro #7, (3NBZ) ClusPro #10, and (3NBZ) FireDock #2. Note that some of these structures overlap with the selected structures from clustering.

**Fig 6:**
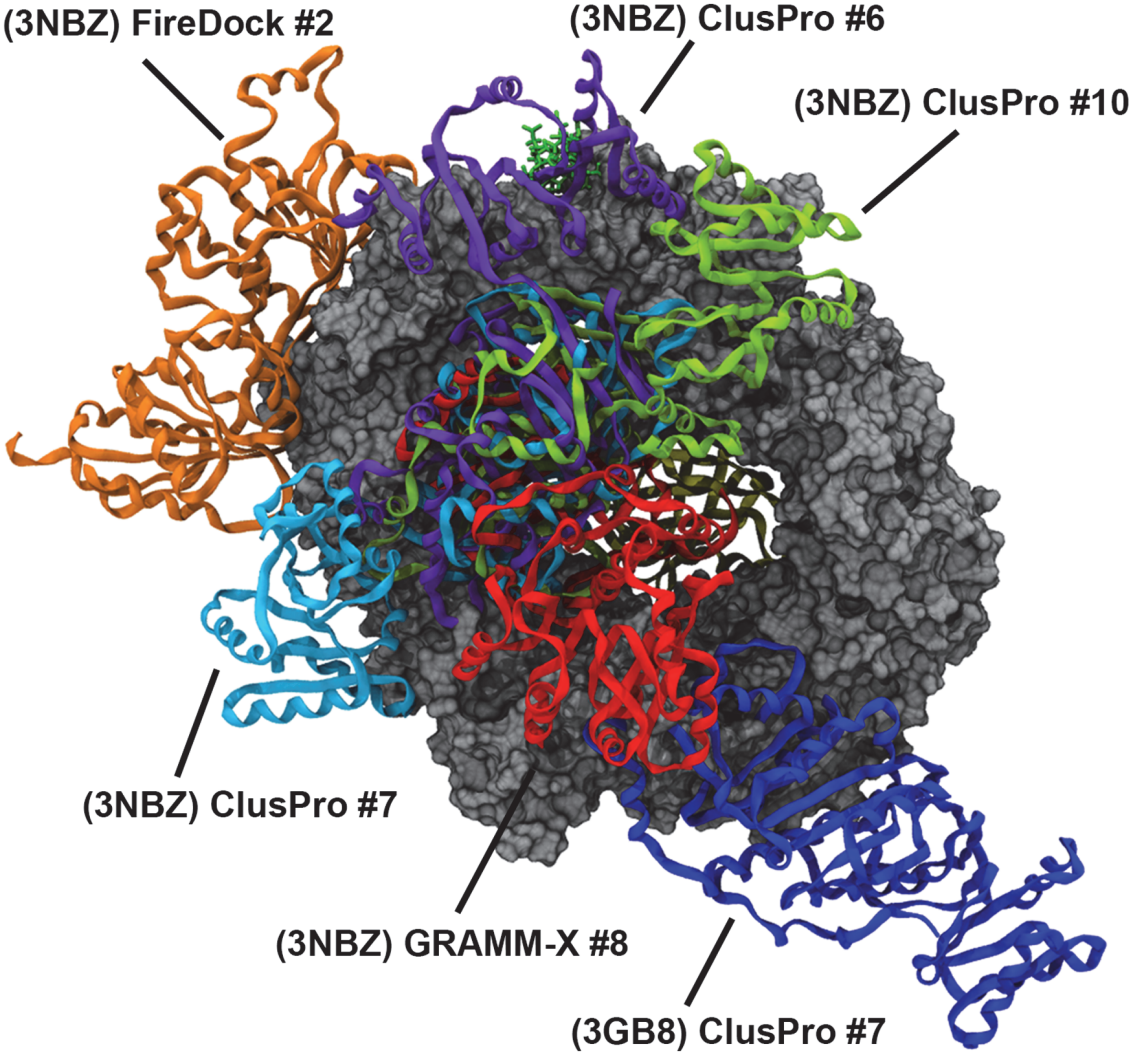
DDX3 molecules from top 6 docked structures overlapped together.

In total, there are 6 docked structures selected for further MD simulation. These binding modes are depicted in Fig. 6 (see S7 Fig. for individual complexes). Of note, DDX3 is located in close proximity to the NES peptide in all but one case (3GB8-ClusPro #7). Additionally, the list of warm and hot interfacial residues on CRM1 and DDX3 are listed in Table 2 for all 6 of these structures.

**Table 2:** List of interfacial warm and hot residues (ΔG_mut→Ala_ > 1kcal/mol) for top 6 docked structures obtained from computational alanine scanning.

Docked Structure	CRM1 Residues (10 ns)	CRM1 Residues (50 ns)	DDX3 Residues (10 ns)	DDX3 Residues (50 ns)
(3NBZ) ClusPro #6	F386* L394* **E510** ^†^ E511 R515^†^ * **R553** ^†^ **R556** ^†^ **R596 R597** ^†^ Q601* V604 N767 D768^†^ **Q853 S857** * T899 N903^†^ E907 L1052	**D401** * **E510** ^†^ R596 **Q895** Q902 Y948	E180* S181* S183 **E196** * L197 R199 E249* M254* **N257** * R259* Y260 R262 R292* S293^†^ * **R294** R296 D474^†^ Q477^†^ * T498^†^ R503^†^ * S520* E523^†^ * **N551** *	E196* L197 M254* R478* R503^†^ *
(3NBZ) ClusPro #7	Y381* **F386** * D401* K446^†^ * **R458** ^†^ E510^†^ **R553** ^†^ R556^†^ R596 Y639 **R765 Q770** E774 I806 K810 S857* F860^†^ Q895 F898 T899 **Q902 N903** ^†^ Q906* **E907**	**F386** * D401* R458^†^ Y463^†^ * R596 **R765** Q902	**E169** * **T171** * R259* **Y260** R263 E332 F340 D354^†^ * D368 **T384** ^†^ * F385^†^ * E388* R394 **D398 R480** ^†^ R534^†^ * N537*	**N173** * R259* **Y260** E388* Q477^†^ * R478*
(3NBZ) ClusPro #10	**Y381** * F386* D447^†^ * K455^†^ **R458** ^†^ E459^†^ * Y463^†^ * E513^†^ R556^†^ K594^†^ R596 **N767** D768^†^ Q770 K810 **Q853** T899 Q902 N903^†^ E907 Y948	Y381* E383* **F386** * Y463^†^ * R596 R597^†^ **R765** N767	K255* **Y260 R263** K264 I310 R311 D329^†^ E332 R333 **K335** D339 **E358** ^†^ * **R362** ^†^ **R363** D368 T369 **K387** * **E388** * **Q390** ^†^ **R394** D398 **R534** ^†^ *	Q225^†^ * Y260 **D368 D398** R488 Y580*
(3NBZ) FireDock #2	N616* D624^†^ E655* L659 L660* **Q663 D666 S667** I669 **Q670** ^*^ **Q671** T673* Q687 **R712** ^*^ **D716** ^†^ ^*^ N719*	L660* Q663 **D666** S667 Q670* T673* **R712** * D716^†^ *	N173* **Q225** ^†^ T384^†^ * **R394** F402^†^ * N414^†^ R488 **D506** ^†^ * N509 K511 **R534** ^†^ * **N537** *	F385^†^ * Q390^†^ Y400 F402^†^ * **R534** ^†^ *
(3NBZ) GRAMM-X #8	K446^†^ * E459^†^ * R596 R765 **K810** F898 **Q902** I1008	R596	W421 E423 K440 D441 **E464** L538 S543^†^ **K564**	R548 K564
(3GB8) ClusPro #7	L67^†^ * **K104** ^†^ E201^†^ **Q990** * D991* **Q993** ^†^ * L996* D1017^†^ * E1024^†^	R62* **W91** ^†^ K104^†^ **Y105** * D196 D991* **Q993** ^†^ *	E249* **R259** * **R276** ^†^ * E277^†^ * Q281^†^ * R287* K288^†^ * Y291* R294 **R296 R311 D312 R315 Y576** *	L197 R276^†^ * **R294** R315 D555* **Y576** *

**bold face** indicates the hot residues (i.e., **ΔG_mut→Ala_** >2 kcal/mol)

* Indicates the residues predicted by SPPIDER.

^†^ Indicates the highly conserved residues based on ConSurf data.

### Extended MD Simulations

The 6 docked structures were selected for extended MD simulation (50 ns) in order to determine if DDX3 could bind CRM1 when placed a distance of 10 Å apart. Note that with the previous 10 ns MD simulations, DDX3 was docked with CRM1, making it unlikely for DDX3 to move far away from CRM1. These extended simulations test more rigorously the binding of DDX3 and the two CRM1 complexes.

**Fig 7:**
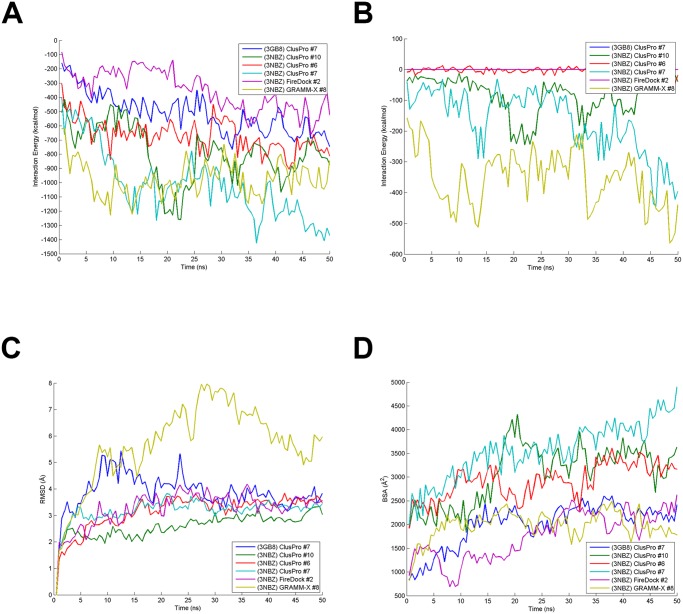
Calculated structural and energetic quantities for extended MD simulation. (A) CRM1-NES-RanGTP—DDX3 interaction energy, (B) RanGTP—DDX3 energy, (C) RMSD, and (D) BSA are plotted over the course of the 50 ns extended MD simulations.

Structural and energetic calculations were performed once again after simulation (Table 3). RMSD and interaction energies indicate that these structures were structurally stable (Fig. 7). Within 10 to 20 ns, all structures’ RMSD plateaued and was maintained until the end of the 50 ns simulation. The interaction energy between CRM1-NES (+/- RanGTP) and DDX3 reached a stable value towards the last 25 ns for all 6 cases, with a few cases exhibiting large oscillation due to some weak binding between certain residues or structural rearrangement (Fig. 7C). Lastly, BSA for each structure was stable for all cases except (3NBZ) ClusPro #7, which increased throughout the 50 ns (Fig. 7D). Interestingly, the interaction energy changed by a non-trivial amount when including interaction between RanGTP and DDX3 (NES-DDX3 interaction was negligible) for three cases: (3NBZ) GRAMM-X #8 (372 kcal/mol), (3NBZ) ClusPro #7 (230 kcal/mol), and (3NBZ) ClusPro #10 (85 kcal/mol). This RanGTP-DDX3 interaction over the 50 ns simulation is shown for each case in Fig. 7B. Note that DDX3 binds on the back of CRM1 where there is an opening allowing for interaction with RanGTP for these three cases.

**Table 3:** Calculated structural and energetic quantities for the top 6 docked structures with rankings after extended MD simulation.

Docked Structure	RMSD (Å)	CRM1-DDX3 Int. Energy (kcal/mol)	(CRM1+RanGTP)-DDX3 Int. Energy (kcal/mol)	MM/GBSA (kcal/mol)	BSA (Å^2^)
(3GB8) ClusPro #7	4.24 (5)	-609.5 (4)	-609.5 (5)	-792.7 (4)	2284.5 (4)
(3NBZ) ClusPro #10	2.92 (1)	-744.0 (2)	-829.1 (3)	-789.6 (5)	3372.1 (2)
(3NBZ) ClusPro #6	3.49 (3)	-716.2 (3)	-719.5 (4)	-809.5 (3)	3137.8 (3)
(3NBZ) ClusPro #7	3.32 (2)	-927.9 (1)	-1157.2 (1)	-817.2 (1)	4005.9 (1)
(3NBZ) FireDock #2	3.55 (4)	-464.5 (6)	-464.5 (6)	-810.7 (2)	2185.0 (5)
(3NBZ) GRAMM-X #8	6.51 (6)	-590.7 (5)	-962.4 (2)	-765.4 (6)	2007.9 (6)

Note: Numbers in parenthesis are the corresponding ranks.

CAS was also performed after the 50 ns simulations for CRM1 and DDX3 (Table 2). In each case, the number of warm/hot residues decreased as compared to the previous 10 ns simulations. This is the result of placing DDX3 from a much greater distance from CRM1 as compared to its location after docking. Generally, residues that appeared in the extended simulations were also present in the 10 ns simulations. Notably, however, (3NBZ) GRAMM-X #8 only had one critical residue on CRM1. This is in line with the data from Table 3 indicating it had among the lowest energetic values and therefore weak binding. Again, its binding was substantially improved through an interaction between RanGTP and DDX3.

The strongest and most stable binding mode of the 6 docked structures based on Tables 2 and 3 is (3NBZ) ClusPro #7. The binding modes of (3NBZ) ClusPro #6 and #10 are very similar to #7, but the DDX3 in #7 is situated in a slightly different orientation such that it has a strong interaction with RanGTP. Data in Table 2 shows individual warm (and hot) interfacial residues according to CAS analysis. Also, highly conserved residues and the ones generally predicted as general hot spots on both sides are annotated in the table. The list of interfacial residues are provided after both post-docking MD and extended MD (after detachment). It is natural that the second list shrinks notably from the original one.

**Fig 8:**
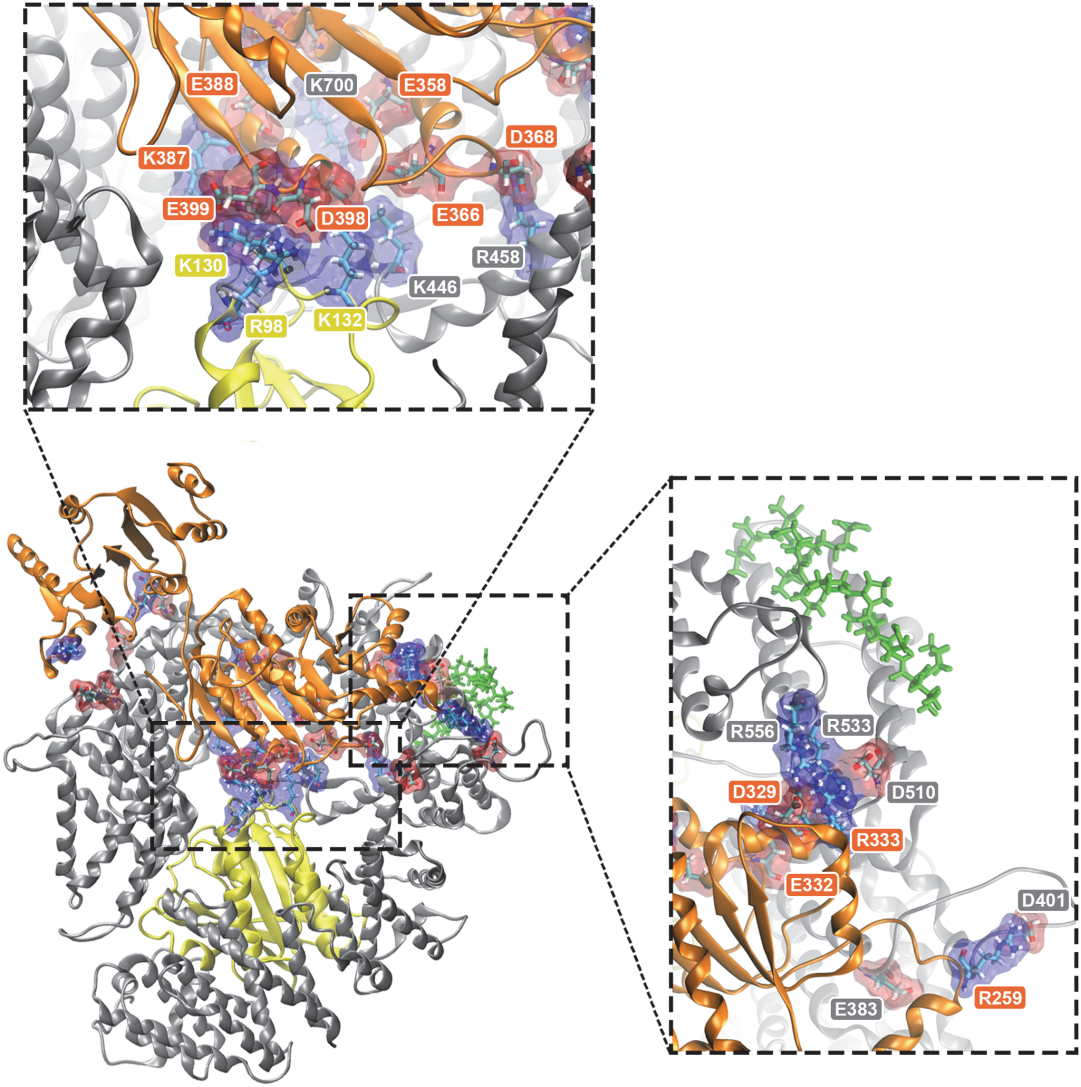
Snapshot of residues at the interface and salt bridge pairs for (3NBZ) ClusPro#7. CRM1 is shown in silver and DDX3 and RanGTP are the orange and yellow ribbon structures, respectively. The green thread is Rev NES. Acidic and basic residues involved in salt bridges are shown in red and blue transparent surfaces, respectively. Top inset shows a zoomed in view of salt bridges around the center of CRM1. Right inset shows zoomed and rotated aerial view of salt bridges near NES region of CRM1. See Table 4 for specific salt-bridge pairs.

Each salt bridge pair for all top 6 docked structures is listed in Table 4. Both (3NBZ) ClusPro #6 and #7 formed 17 salt bridges between CRM1 and DDX3 throughout the time evolution of the 50 ns simulation, but #7 also formed the most salt bridges between DDX3 and RanGTP with a total of 6. It shows the importance of DDX3-RanGTP binding in some of the most promising complex formation modes. The formed salt bridges are shown in Fig. 8 for (3NBZ) ClusPro #7 as the top candidate. Also, the bridge distances are plotted throughout the simulation in S8 Fig. Most of them appear either in the central CRM1 hole (in conjunction with RanGTP and CRM1) or in the vicinity of NES binding cleft (see the insets of Fig. 8). These areas of salt bridge concentration act like anchors to maintain the binding between DDX3 and CRM1.

**Table 4:** Summary of salt bridge pairs formed throughout extend MD simulation.

(3NBZ) ClusPro #6				(3NBZ) ClusPro #7				(3NBZ) ClusPro #10			
DX3	CRM1	DDX3	RanGTP	DDX3	CRM1	DDX3	RanGTP	DDX3	CRM1	DDX3	RanGTP
E180	R553	—		R259	E383	K387	D128	R259	E774	D354	K134
E184	R485			R259	D401	R394	D128	R263	E907	E366	K127
E189	R765			D329	R556	D395	K132	R333	E907		
E196	R556			E332	R553	D398	R95	R333	E908		
E196	K594			R333	E510	D398	K130	E358	K446		
E196	R596			D350	R765	E399	K130	D368	R765		
196	R597			D354	R765			D368	K810		
R202	E510			E358	K700			E388	K455		
E216	R382			E366	K446			D395	K446		
R259	E955			D368	R458			D398	R596		
R263	E955			E388	K700			D398	R597		
R292	D768			E388	R765			E399	R556		
E449	R515			D395	K446			R534	D401		
K451	D512			R480	E907						
R478	D401			R480	E908						
R503	E510			R534	E774						
K451	E511			R534	E818						

(3NBZ) FireDock #2				(3NBZ) GRAMM-X #8				(3GB8) ClusPro #7			
DDX3	CRM1	DDX3	RanGTP	DDX3	CRM1	DDX3	RanGTP	DDX3	CRM1	DDX3	RanGTP
R394	D624	—		E423	K446	E423	K132	E189	K54	—	
E413	K674			E423	K455	E423	K134	R276	D196		
K440	E774			E424	R382	E547	K132	R294	D991		
K440	D782			D426	K446	E547	K134	D305	K192		
R488	E651			D426	K455	E577	K99	R311	D1017		
R488	E655			K440	D768			R315	D1017		
K491	E651			K452	E510			D555	R62		
K491	E774			D459	R556			D558	K104		
R534	D666			E464	R596			K564	D152		
R534	D716			E464	R765			E572	K112		
				K564	E955			E577	K112		
				K564	D1007						
				E572	K1012						
				E572	R1016						
				E577	K1012						
				E577	R1016						

### Flexible Multi-Domain Docking

The two domains of DDX3 are separated by a highly flexible loop that allows it to take open or close conformations. Meanwhile, DDX3 can be in a different state other than the open conformation when encountering CRM1. Other possible conformations must therefore be considered. Ideally, the docking algorithm should take the highly flexible structure of such proteins into account. Not all of the available docking tools are capable of this task. Moreover, even servers with this feature do not guarantee satisfactory results, especially in case of missing residues in the flexible loop region. To account for DDX3 flexibility and as a complement to the main protocol, we applied the flexible multi-domain docking (FMD) utility available in HADDOCK [56] (see Methods for details).

The FMD scheme was carried out for DDX3 on both 3GB8 and 3NBZ structures. The top 200 solutions were grouped into 4 and 10 clusters for 3GB8 and 3NBZ, respectively. The results were examined to ensure feasibility and structural integrity. Cases containing a non-physical DDX3 state (i.e. highly twisted or separated domains) were rejected. Also, overlap with RanGTP and RanBP1 binding site led to their rejection. After examining all candidates, only one of the results from the FMD application on 3GB8 (CRM1 without RanGTP) was selected while none of 3NBZ results (CRM1 bound to RanGTP) proved feasible. In the selected case, binding occurs far from Rev binding cleft at CRM1 terminal domains (see S9 Fig.). This mode is quite similar to the top 3GB8 candidate obtained from our hybrid protocol. The index of hot interfacial residues based on CAS is listed in S2 Table. Although the FMD method offers a powerful framework for examining multi-domain proteins, obtaining promising results is not always an easy procedure. This may be more relevant in cases with missing residues in the linker region.

## Discussion

Nucleocytoplasmic traffic across the nuclear envelop is regulated by the nuclear pore complex (NPC) [60]. The human immunodeficiency virus (HIV) exploits the nucleocytoplasmic pathway to export its RNA transcripts across the NPC to the cytoplasm. The HIV relies on a single host protein, CRM1, to export its unspliced and partially spliced RNA transcripts. Recent findings have implicated a DEAD-box helicase, DDX3, in HIV replication and a member of the export complex, making it an appealing target for anti-HIV drug inhibition.

Computational modeling approaches have offered a powerful platform for predicting the quantitative biology of nucleocytoplasmic transport processes [61–65]. In the present work, we used a hybrid computational protocol consisting of preliminary docking, MD equilibration, approximate free energy calculation, clustering and computational alanine scanning accompanied by evolutionary analysis and protein-protein binding prediction to investigate the binding of DDX3 to CRM1 in the context of HIV-1 Rev-mediated nuclear export of partially spliced and unspliced HIV-1 RNA. While it has yet to be tested, our hybrid computational protocol holds promise as a general approach to discover binding modes for protein-protein complexes for which structural experimental data are missing. Future work will need to validate this protocol by using a range of protein-protein complexes whose binding modes have been previously determined.

We used multiple docking methods and gathered the obtained results in a single pool of candidates. We then post-processed and refined the results using MD simulations. While most docking tools use some criteria obtained from a single sample point, MD trajectories enable us to achieve multi-point sampling as well as conformational equilibration. MM/GBSA, structural stability, interaction energy and BSA were used to re-rank different complexes. Our results showed a substantial change in the rankings after MD equilibration. Then, the docked complexes were clustered based on the proximity of the ligands. The strongest members of the most populated clusters were selected as the representatives of the whole pool of candidates. Top-ranked complexes were again equilibrated after deliberate separation of ligand from the host to examine the binding reconstruction and stability.

Included in our system are Rev-NES and RanGTP. Using three high performance docking approach (ClusPro2.0, FireDock and GRAMM-X), for each of the two types of host (w/ and w/o RanGTP from 3NBZ and 3GB8 respectively) the best 10 candidates were selected from each docking server and were merged together in a pool of 60. While DDX3 molecules spread all around CRM1 without RanGTP, addition of RanGTP lead to higher accumulation of ligands on the back side of CRM1 (opposite side of the RanGTP binding site). This attractive area is also in proximity of NES for DDX3, which would place DDX3 in a favorable position for interaction with Rev and HIV-1 RNA. After performing MD equilibration, new rankings were obtained based on approximate free energy and structural stability. According to the relative strength of all 60 candidates, ClusPro2.0 produced the strongest complexes in all cases, both with and without RanGTP. Considering all docking tools together, MM/GBSA values are not meaningfully different between the two cases (with and without RanGTP). However ClusPro2.0 candidates have higher MM/GBSA with RanGTP. Also, interaction energy, RMSD and BSA showed their best values among ClusPro2.0 results in the presence of RanGTP. Hence, the most favorable cases were achieved by ClusPro2.0 and in the presence of RanGTP. DDX3 binding sites without RanGTP seem to be distinct from the host with RanGTP. It could be that DDX3 can bind without RanGTP erroneously; nonetheless, this structure may not leave the nucleus. Instead, the correct binding spot for DDX3 (correct as in capable of allowing export) may be present once RanGTP has bound to CRM1.

Computational alanine scanning of interfaces in all binding candidates helped us determine the distribution of hot interfacial residues collected from the post-processed docking analysis. The CAS results corroborated the observation mentioned about the DDX3 binding mode distribution all around CRM1 in different cases. In addition, conservation analysis and potential hot residue prediction were performed to accompany the main results. However, comparing CAS hot residue distribution with the outcome of evolutionary analysis, we could not see a strong correlation and agreement between them. The only region that appeared to be hot in all three distributions is the region on the back side of CRM1 neighboring the NES binding cleft (Figs. 4 and 5). Indeed, any region closer to the cargo-binding site is essential for CRM1’s role and must be the most conserved and active domain.

Clustering of the docking solutions helped us find the most attractive regions for accumulation of binding mode candidates. Together with the strongest binding modes, 6 top candidates were selected, wherein 5 had RanGTP in the host structure. Four DDX3 molecules were bound to CRM1 on the site opposite RanGTP, with some of these DDX3 molecules being able to interact with RanGTP through the CRM1 central hole. One DDX3 molecule sits on the outer rim close to NES binding cleft and the last one adheres to the terminal domains. After a 10Å separation for the 50ns MD simulation, the top 6 candidates could again establish stable, strong binding. For the best 2 in the top 6 ((3NBZ) ClusPro #7 and (3NBZ) GRAMMX #8), it was observed that interaction with RanGTP played an important role in stabilizing the binding. In agreement with previous research [13], DDX3 and CRM1 can bind stably in the absence of RanGTP, but the addition of RanGTP leads to more robust interactions. As previously mentioned, the most attractive binding area for DDX3 was the back side of CRM1, close to NES binding site. Of the docked structures that exhibited binding of DDX3 in this region, the strongest binding modes also included interaction between DDX3 and RanGTP. Some of these binding modes, specifically (3NBZ) ClusPro #7 and (3NBZ) GRAMMX #8, became the strongest modes because they exhibited salt bridge formation between DDX3 and RanGTP.

The order of events occurring during the formation and transport of HIV mRNA export complex is not fully understood. Our results suggest that the binding of DDX3 is stronger and more directed toward a specific region in the presence of RanGTP. Given that CRM1 does not leave the nucleus without bound RanGTP, these data could suggest that DDX3 binds to CRM1 in the nucleus and is kept bound throughout the export process to be utilized as an RNA helicase down the road.

On the other hand, DDX3 has only been shown to be involved in CRM1-mediated nuclear export during HIV-1 infection. HIV-1 Rev may form some transitory interaction with DDX3 and deposit it in the vicinity of CRM1. In other words, the interaction of DDX3 with CRM1 cannot rely on free diffusion, but if it can be held in the vicinity of CRM1 for sufficient time to sample different conformations, then stable binding with CRM1 can be achieved long enough to last throughout the transport process. In an alternative scenario, when RanGTP is present, the affinity of CRM1 toward Rev-NES is increased, and if DDX3 is bound to Rev, DDX3 can be re-localized within the vicinity of the NES-binding site of CRM1. This may explain why in the presence of RanGTP-DDX3 binding sites are so focused instead of spread all over CRM1.

## Supporting Information

S1 Fig Superposition of the top 10 (for each host structure) GRAMM-X docked structures. The structures are shown for (A) 3NBZ (w/RanGTP) back side (B) 3NBZ front side, and (C) 3GB8 (w/o RanGTP) back side, and (D) 3GB8 front side. CRM1 is shown in silver and RanGTP is the yellow ribbon structure.
(TIF)

S2 Fig Superposition of the top 10 (for each host structure) FireDock docked structures. The structures are shown for (A) 3NBZ (w/RanGTP) back side (B) 3NBZ front side, and (C) 3GB8 (w/o RanGTP) back side, and (D) 3GB8 front side. CRM1 is shown in silver and RanGTP is the yellow ribbon structure.
(TIF)

S3 Fig Calculated structural and energetic quantities for the 60 docked structures. Bar graphs show interaction energy, MM/GBSA, RMSD and BSA for complexes (A) without and (B) with RanGTP.
(TIF)

S4 Fig Selected samples of MD simulation results. (TIF)

S5 Fig Highlighted predicted interfacial residues from SPPIDER. Highlighted structures are (A) CRM1 (3NBZ), (B) CRM1 (3GB8), and (C) 2I4I.
(TIF)

S6 Fig Evolutionary conserved residues highlighted based on score from ConSurf. Scale is from 1 (blue) to 9 (red). Structures highlighted are (A) CRM1 (3NBZ), (B) CRM1 (3GB8), and (C) 2I4I.
(TIF)

S7 Fig Top 6 selected candidates shown separately. (TIF)

S8 Fig Salt bridge distances vs. time. Salt bridges formed throughout the extended MD trajectory of (3NBZ) ClusPro #7 between DDX3 and CRM1. Graphs are of salt bridges (A) present and (B) absent as hotspots in CAS analysis. First and second residues in the salt bridge pair (see graph legend) are from DDX3 and CRM1, respectively.
(TIF)

S9 Fig Top candidate obtained from flexible multi-domain docking. (TIF)

S1 Table Average values of the calculated structural and energetic quantities for the 60 docked structures during MD post-processing phase. Mean values of RMSD, interaction energy, MM/GBSA and BSA are listed in the table for different tested host, as well as each docking server.
(DOCX)

S2 Table List of interfacial warm and hot residues (ΔG_mut→Ala_ > 1kcal/mol) for top FMD binding mode obtained from computational alanine scanning. **bold face** indicates the hot residues (i.e., **ΔG**_**mut→Ala**_ >2 kcal/mol).
(DOCX)
