# Supplementary figures and images for: The Interaction of RNA Helicase DDX3 with HIV-1 Rev-CRM1-RanGTP Complex during the HIV Replication Cycle
Source: PLoS One. 2015 Feb 27;10(2):e0112969. doi: 10.1371/journal.pone.0112969 (PMC4344243; doi:10.1371/journal.pone.0112969)

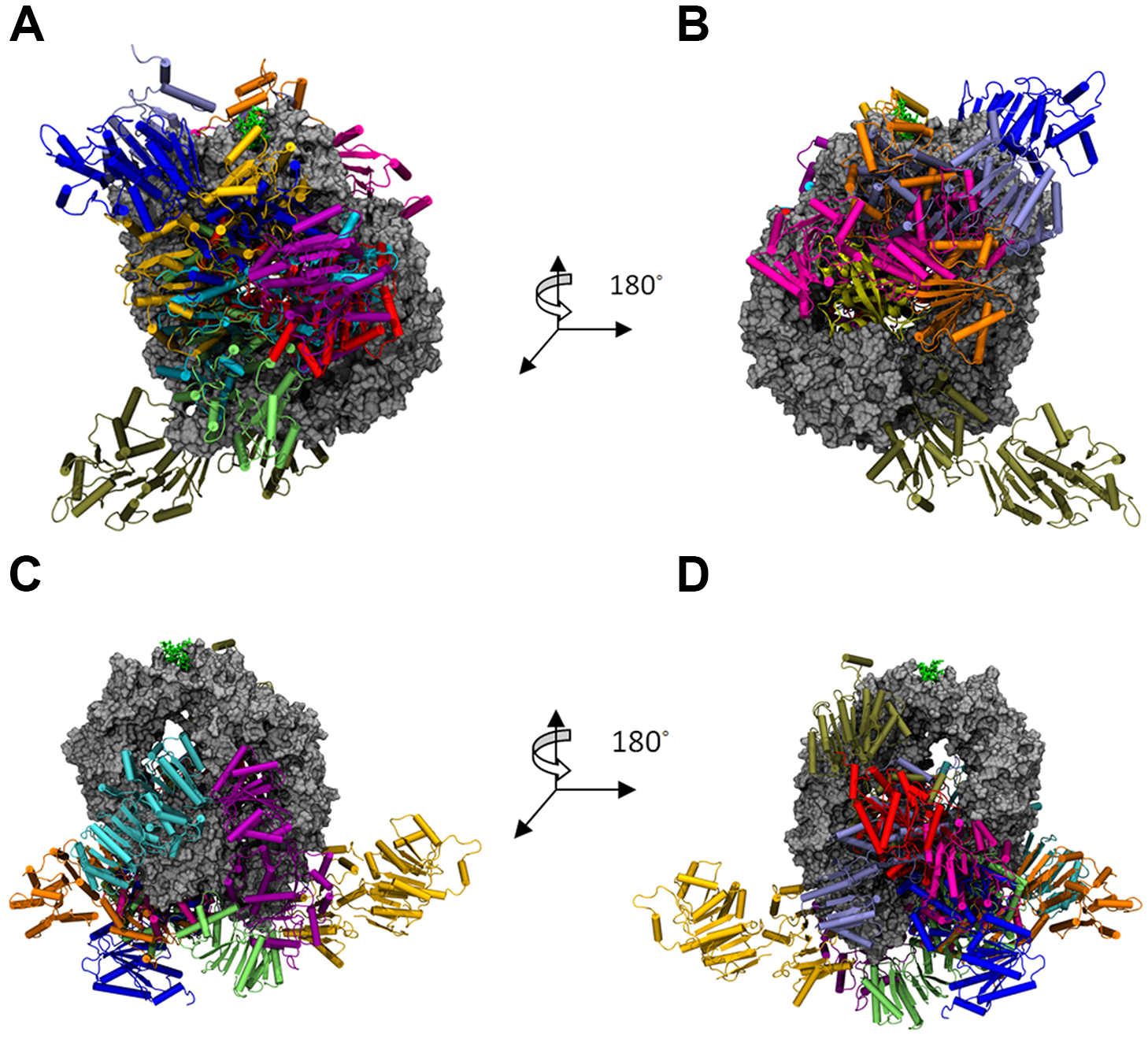

Supplement: S1 Fig — The structures are shown for (A) 3NBZ (w/RanGTP) back side (B) 3NBZ front side, and (C) 3GB8 (w/o RanGTP) back side, and (D) 3GB8 front side. CRM1 is shown in silver and RanGTP is the yellow ribbon structure. (TIF) [file pone.0112969.s001.tif]

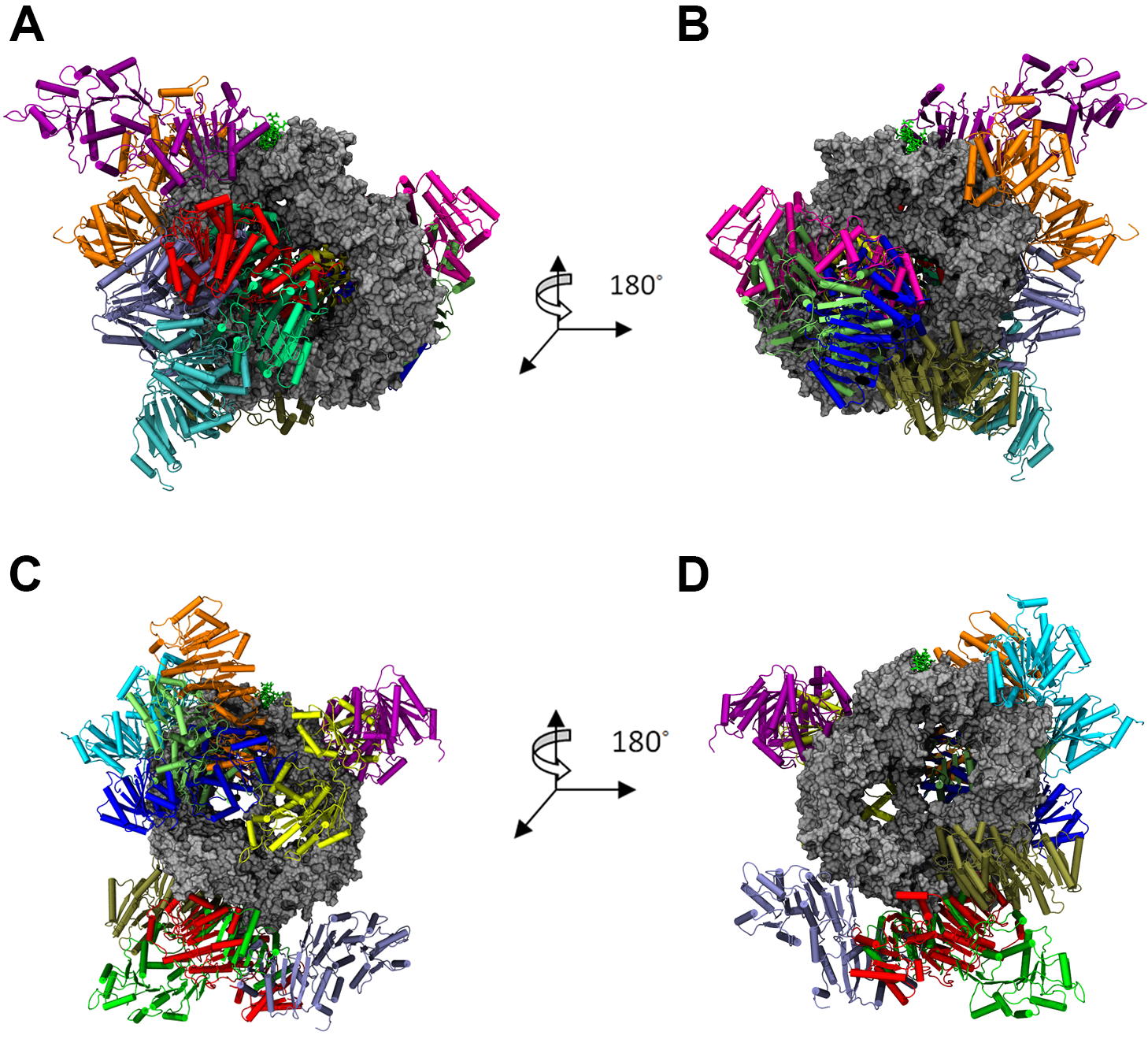

Supplement: S2 Fig — The structures are shown for (A) 3NBZ (w/RanGTP) back side (B) 3NBZ front side, and (C) 3GB8 (w/o RanGTP) back side, and (D) 3GB8 front side. CRM1 is shown in silver and RanGTP is the yellow ribbon structure. (TIF) [file pone.0112969.s002.tif]

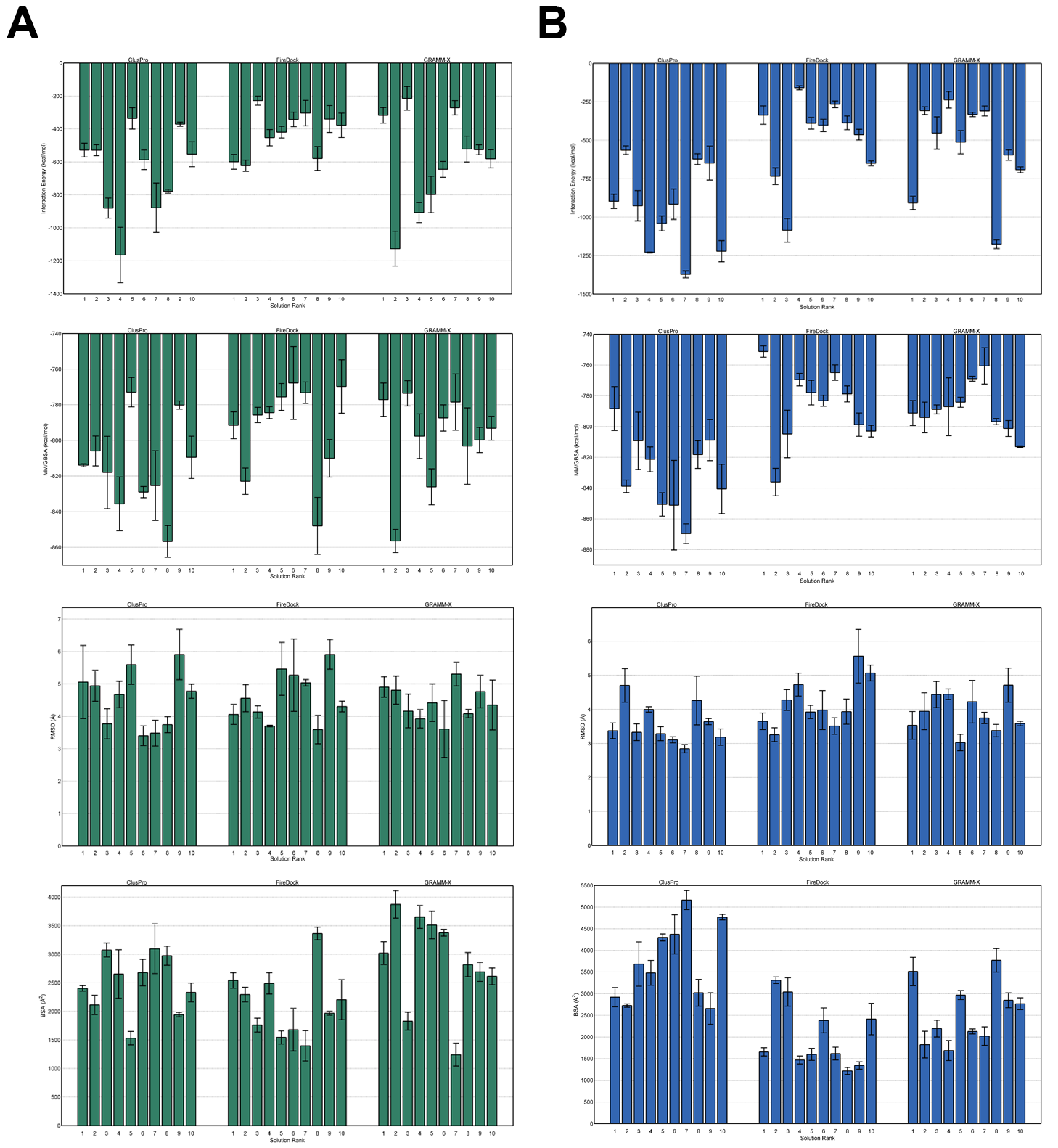

Supplement: S3 Fig — Bar graphs show interaction energy, MM/GBSA, RMSD and BSA for complexes (A) without and (B) with RanGTP. (TIF) [file pone.0112969.s003.tif]

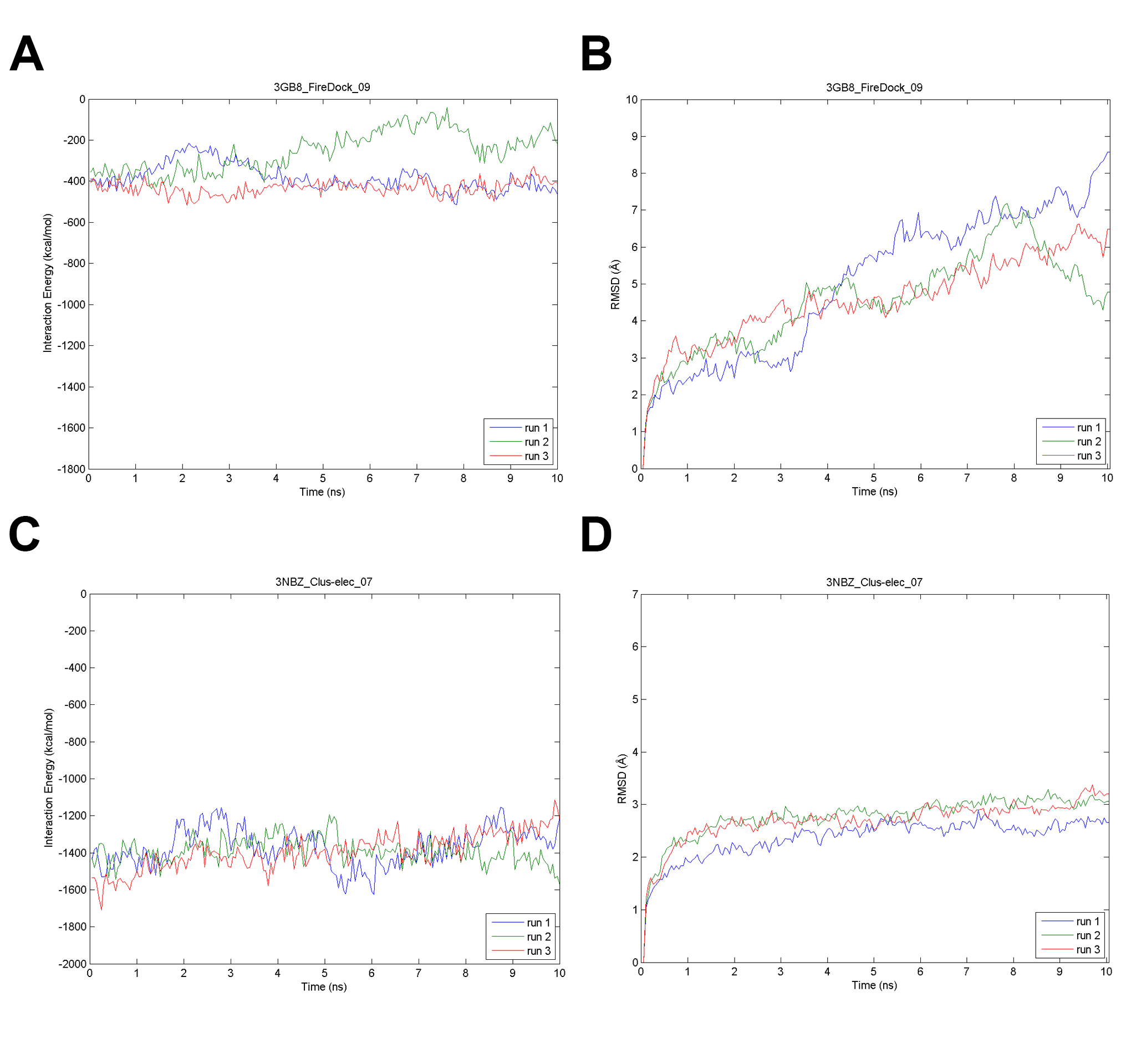

Supplement: S4 Fig — (TIF) [file pone.0112969.s004.tif]

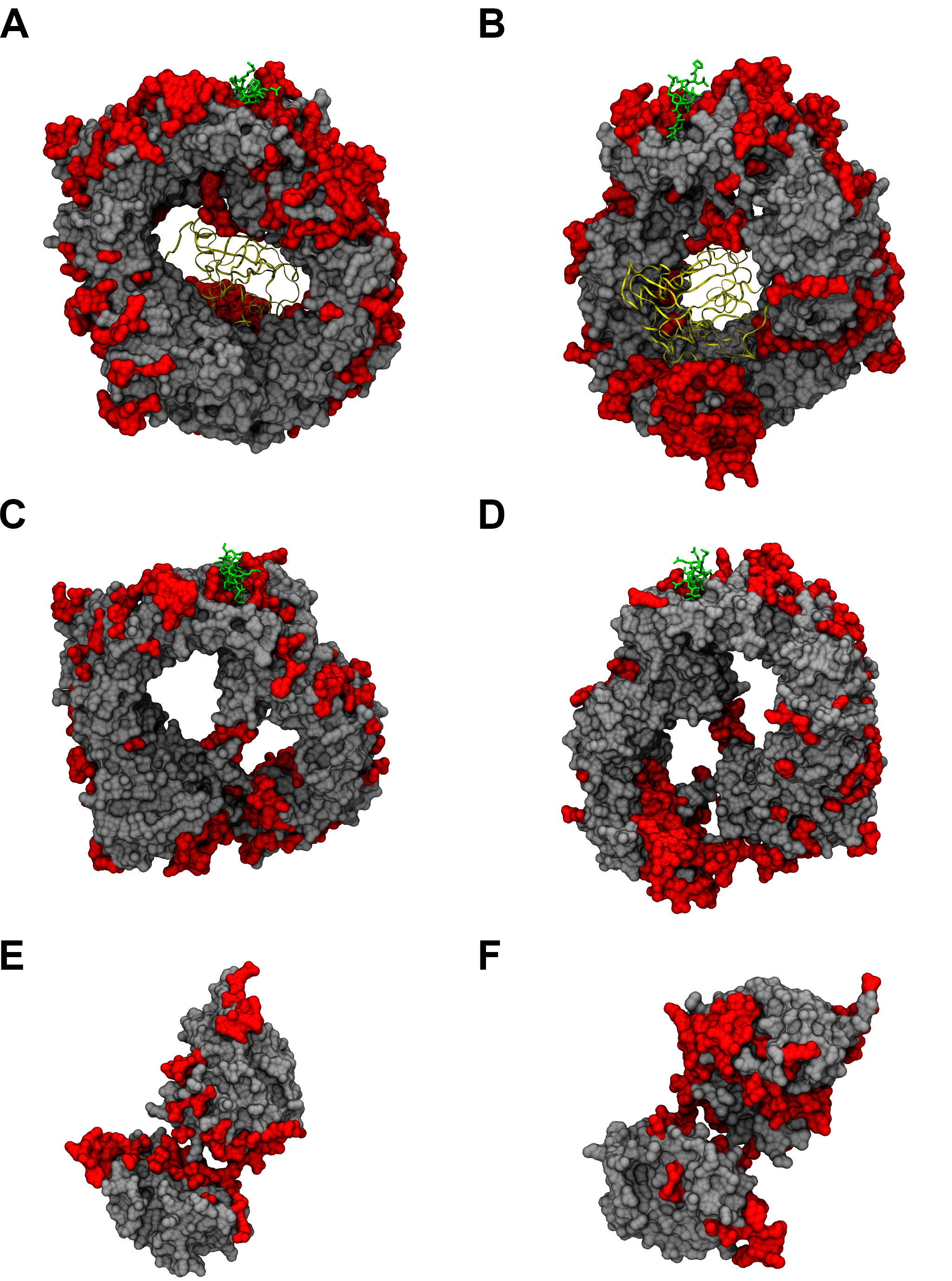

Supplement: S5 Fig — Highlighted structures are (A) CRM1 (3NBZ), (B) CRM1 (3GB8), and (C) 2I4I. (TIF) [file pone.0112969.s005.tif]

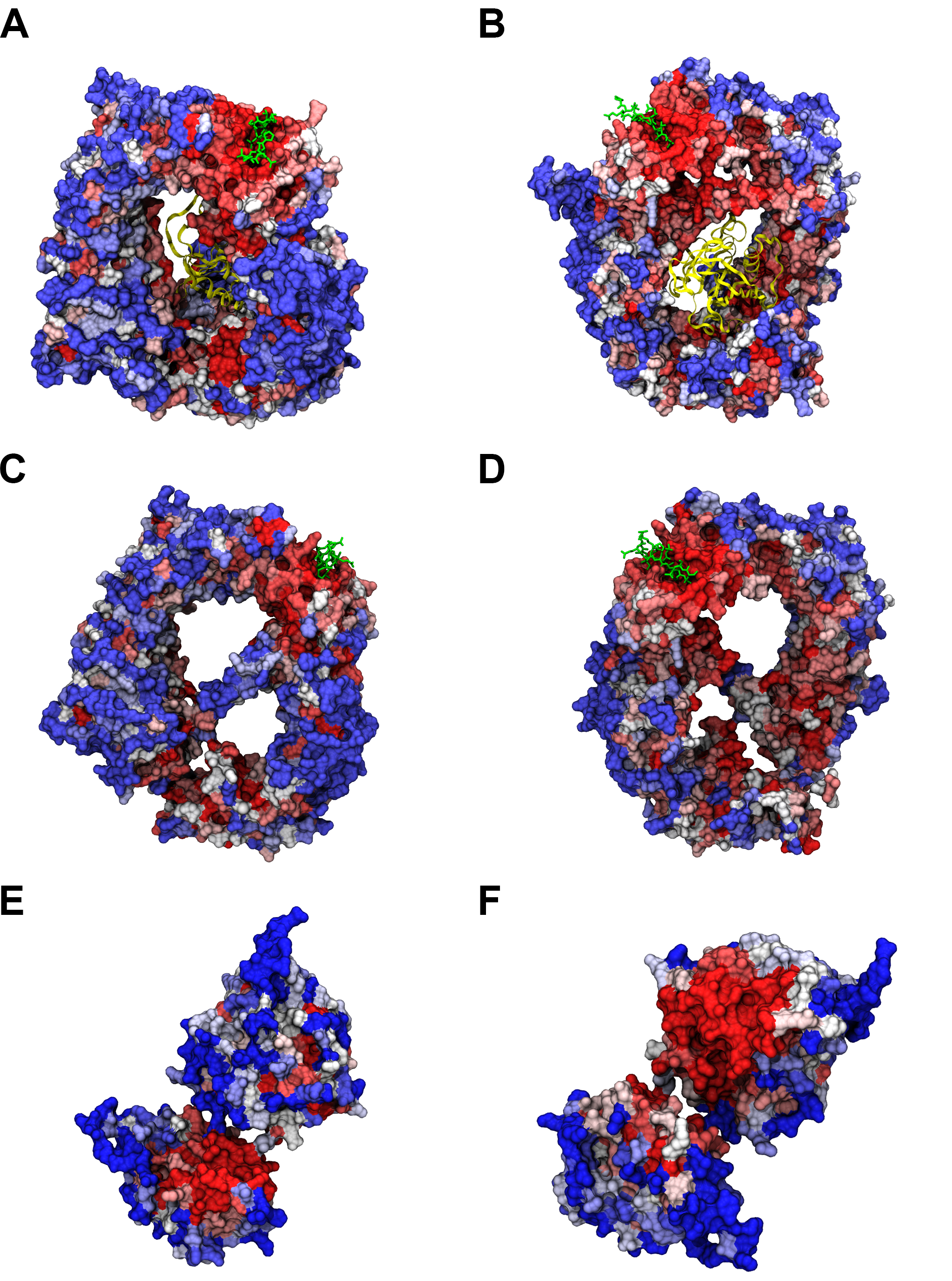

Supplement: S6 Fig — Structures highlighted are (A) CRM1 (3NBZ), (B) CRM1 (3GB8), and (C) 2I4I. (TIF) [file pone.0112969.s006.tif]

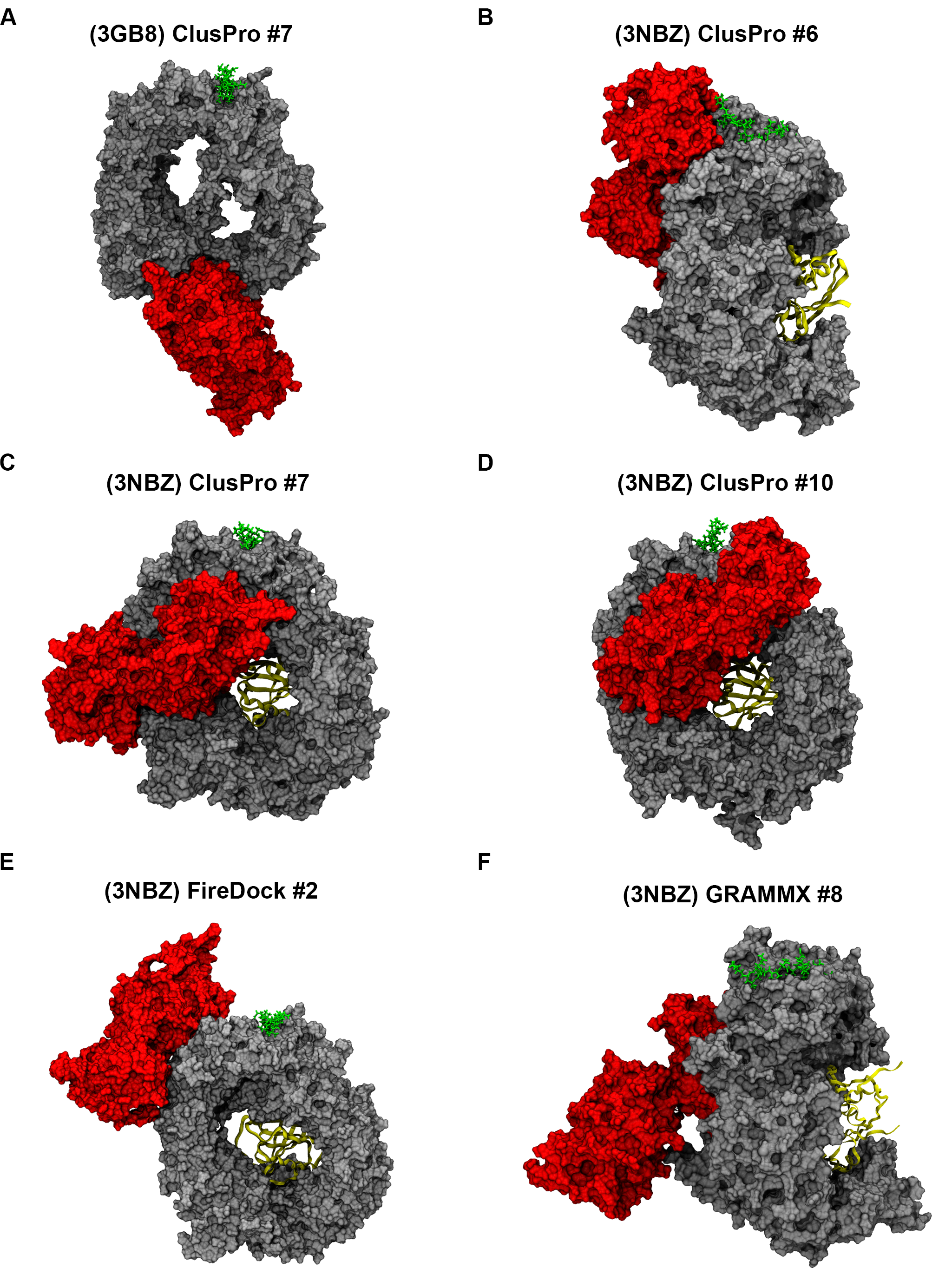

Supplement: S7 Fig — (TIF) [file pone.0112969.s007.tif]

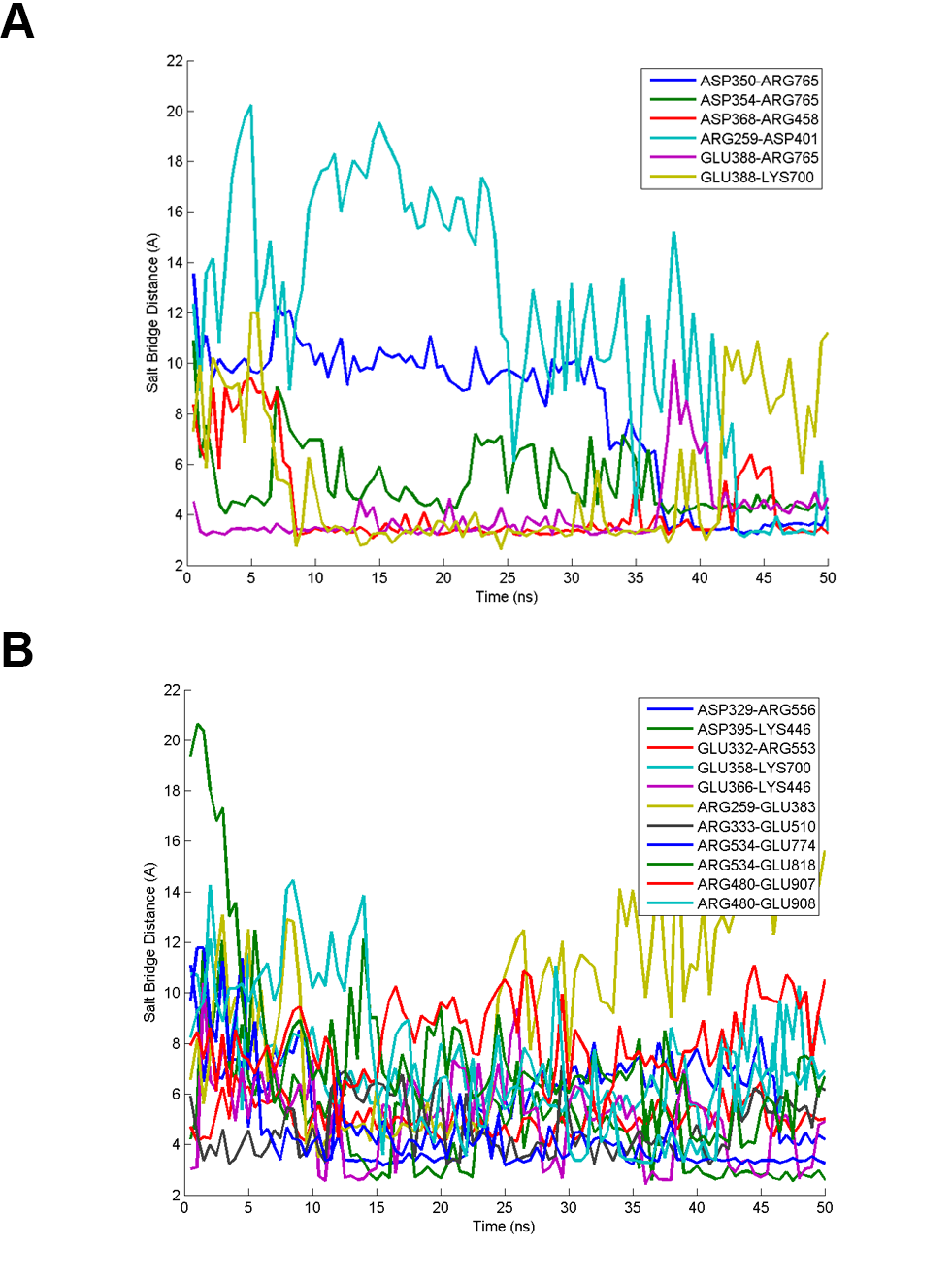

Supplement: S8 Fig — Salt bridges formed throughout the extended MD trajectory of (3NBZ) ClusPro #7 between DDX3 and CRM1. Graphs are of salt bridges (A) present and (B) absent as hotspots in CAS analysis. First and second residues in the salt bridge pair (see graph legend) are from DDX3 and CRM1, respectively. (TIF) [file pone.0112969.s008.tif]

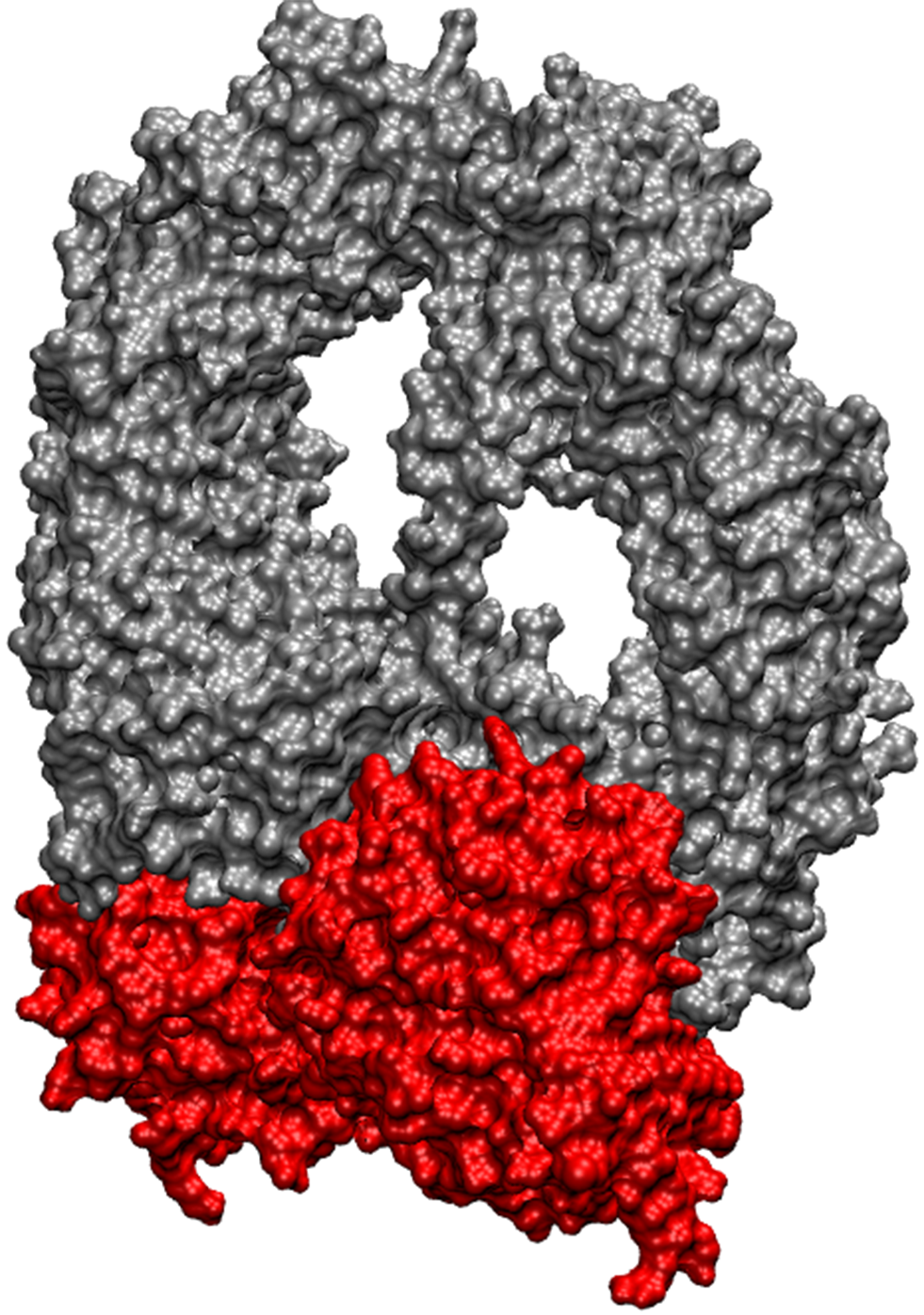

Supplement: S9 Fig — (TIF) [file pone.0112969.s009.tif]
