# Supplementary material for: The Interaction of RNA Helicase DDX3 with HIV-1 Rev-CRM1-RanGTP Complex during the HIV Replication Cycle
Source: PLoS One. 2015 Feb 27;10(2):e0112969. doi: 10.1371/journal.pone.0112969 (PMC4344243; doi:10.1371/journal.pone.0112969)
Supplement: S1 Table — Mean values of RMSD, interaction energy, MM/GBSA and BSA are listed in the table for different tested host, as well as each docking server. (DOCX) [file pone.0112969.s010.docx]

**Table S1**

| **Server** | **RMSD (Å)** | **Int. Energy (kcal/mol)** | **MM/GBSA (kcal/mol)** | **BSA (Å^2^)** |
| --- | --- | --- | --- | --- |
| ClusPro | 3.57 ± 0.58 | -944.1 ± 276.5 | -829.7 ± 24.7 | 3707.8 ± 897.0 |
| FireDock | 4.19 ± 0.73 | -488.0 ± 269.1 | -786.8 ± 24.4 | 2004.0 ± 735.8 |
| GRAMM-X | 3.90 ± 0.54 | -552.9 ± 300.7 | -788.6 ± 15.1 | 2570.9 ± 713.1 |
| 3NBZ average: | 3.88 ± 0.65 | -661.7 ± 341.0 | -801.7 ± 29.2 | 2760.9 ± 1046.3 |
| ClusPro | 4.53 ± 0.89 | -660.8 ± 259.0 | -814.7 ± 24.9 | 2479.7 ± 516.1 |
| FireDock | 4.60 ± 0.78 | -426.9 ± 134.9 | -792.9 ± 26.3 | 2123.9 ± 586.8 |
| GRAMM-X | 4.43 ± 0.51 | -591.3 ± 290.5 | -799.3 ± 25.3 | 2862.8 ± 826.1 |
| 3GB8 average: | 4.52 ± 0.72 | -559.6 ± 250.2 | -802.3 ± 26.3 | 2488.8 ± 703.9 |
| Overall average: | 4.20 ± 0.75 | -610.7 ± 300.9 | -802.0 ± 27.5 | 2624.8 ± 894.7 |
