# Supplementary material for: The Interaction of RNA Helicase DDX3 with HIV-1 Rev-CRM1-RanGTP Complex during the HIV Replication Cycle
Source: PLoS One. 2015 Feb 27;10(2):e0112969. doi: 10.1371/journal.pone.0112969 (PMC4344243; doi:10.1371/journal.pone.0112969)
Supplement: S2 Table — bold face indicates the hot residues (i.e., ΔG mut→Ala >2 kcal/mol). (DOCX) [file pone.0112969.s011.docx]

**Table S2**

| **CRM1 Residues** | **DDX3 Residues** |
| --- | --- |
| **K54 D64** T65 N72 Y78 R90 R96 N97 Q98 H145 **H193** N200 N980 Q990 Q993 **L996 Q1021** | R199 **R202** Q225 T226 S228 Q281 F402 **R503 E523** E524 N551 K554 **Y576** E577 **H578 H579** |
